# Effect of composite biodegradable biomaterials on wound healing in diabetes

**DOI:** 10.3389/fbioe.2022.1060026

**Published:** 2022-11-25

**Authors:** Sihang Ren, Shuaichen Guo, Liqun Yang, Chenchao Wang

**Affiliations:** ^1^ NHC Key Laboratory of Reproductive Health and Medical Genetics (Liaoning Research Institute of Family Planning), The Affiliated Reproductive Hospital of China Medical University, Shenyang, China; ^2^ Department of Plastic Surgery, The First Hospital of China Medical University, Shenyang, China; ^3^ The First Clinical College of China Medical University China Medical University, Shenyang, China; ^4^ Department of Plastic Surgery, The Second Hospital of Dalian Medical University, Dalian, China

**Keywords:** biodegradable biomaterials, diabetic wound, drug delivery systems (DDS), mesenchymal stem cells (MSCs), exosomes

## Abstract

The repair of diabetic wounds has always been a job that doctors could not tackle quickly in plastic surgery. To solve this problem, it has become an important direction to use biocompatible biodegradable biomaterials as scaffolds or dressing loaded with a variety of active substances or cells, to construct a wound repair system integrating materials, cells, and growth factors. In terms of wound healing, composite biodegradable biomaterials show strong biocompatibility and the ability to promote wound healing. This review describes the multifaceted integration of biomaterials with drugs, stem cells, and active agents. In wounds, stem cells and their secreted exosomes regulate immune responses and inflammation. They promote angiogenesis, accelerate skin cell proliferation and re-epithelialization, and regulate collagen remodeling that inhibits scar hyperplasia. In the process of continuous combination with new materials, a series of materials that can be well matched with active ingredients such as cells or drugs are derived for precise delivery and controlled release of drugs. The ultimate goal of material development is clinical transformation. At present, the types of materials for clinical application are still relatively single, and the bottleneck is that the functions of emerging materials have not yet reached a stable and effective degree. The development of biomaterials that can be further translated into clinical practice will become the focus of research.

## 1 Introduction

Diabetes (DM) is a chronic disease that is difficult to treat. It is estimated that the global number of patients will reach 592 million by 2035 (Guariguata et al., 2014). Foot ulcers affect 6.3% of diabetes patients in the world (Zhang P. et al., 2017), and the national health service of the United Kingdom spent 580 million pounds every year to treat patients with related diseases (Kerr et al., 2019). In addition, foot ulcers significantly impact patients’ quality of life, leading to more pain, less vitality, and social function limitations (Meaume et al., 2017). With the increase in the prevalence of diabetes, wounds with poor healing or nonhealing have become a serious global health problem. Hyperglycemia can impair wound healing through different mechanisms.

Hypoxia is a major cause of diabetic wound damage caused by two factors: limited oxygen supply and high oxygen consumption in the wound. Neurological sensory loss may aggravate traumatic tissue loss, while epithelium delays wound healing due to cell proliferation and resistance to growth factors (Tuhin et al., 2017). The imbalance between angiogenic factors and vascular inhibitory factors leads to the limitation of feeding sources (Gadelkarim et al., 2018). The high oxygen consumption of activated inflammatory cells makes this dilemma even more difficult. In this environment, the functions of various wound healing-related cells, such as keratinocytes, fibroblasts, and vascular endothelial cells, are inhibited (Bai et al., 2020). Studies have shown that diabetes wounds have higher activity of matrix metalloproteinases (MMPs) than healthy wounds (Louiselle et al., 2021). Therefore, collagen fibers are destroyed faster than their secretion, delaying the formation of sufficient granulation tissue (Pan et al., 2022). The lack of nutrition in the wound is more serious, and the main culprit is bacterial colonization (Amirrah et al., 2020).

The standard treatment of diabetes wounds includes wound cleaning, revascularization, infection control, blood sugar control, foot care, and limb lifting. However, these treatments are often insufficient to ensure good wound healing, and even after standardized treatment, patients still face the possibility of amputation (Shettigar and Murali, 2020). Unfortunately, the simple application of biomaterials is still difficult to achieve absolute benefits for wound healing and even produce slight adverse effects due to the characteristic of their degradation products. The idea of wound healing with biomaterials as the core and additional active ingredients is advanced and desirable. Therefore, the idea of using mesenchymal stem cells, various drugs, and active factors combined with biodegradable biopolymer materials to treat refractory wounds came into being. Through a comprehensive literature search of published and ongoing studies, we aim to provide an overview of the experimental basis, scientific background, and possible clinical applications, to clarify the therapeutic role of composite degradable biomaterials combined with multiple preparation methods in diabetic wound healing.

## 2 Clinical strategies for diabetes wound treatment

**FIGURE 1:**
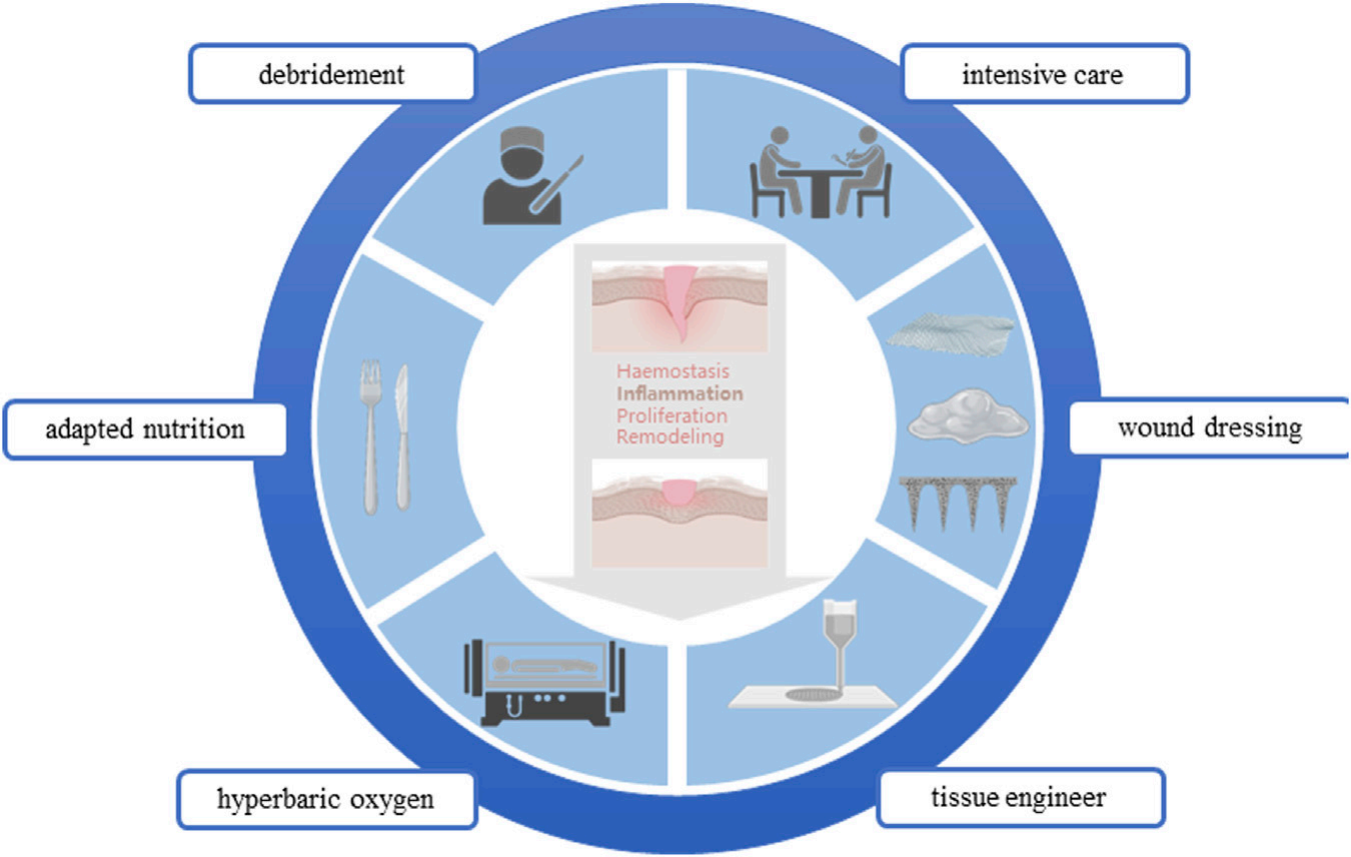
Clinical strategies for diabetic wounds.

In the healing treatment of diabetes wound, the core problem is that the time axis of wound repair is disturbed, which makes the wound healing process fall into the inflammatory stage, and the vascularization is damaged, hypoxia, immune cell dysfunction, and then induce the inactive tissue to provide a suitable environment for bacterial growth and biofilm formation. And then aggravate the inflammatory reaction, and inhibit ECM deposition and tissue repair. And ultimately lead to wound nonhealing, amputation, and even life-threatening (Saghazadeh et al., 2018). The treatment of diabetes wound needs to consider many factors. In general, it can be broadly divided into systemic treatment and local treatment. In addition to actively treating the primary disease, the systemic treatment also needs to adjust the nutritional structure and improve the nutritional status; The local treatment gives priority to the repair of local wounds, and the etiological treatment can be carried out from the four stages of wound repair. In clinical practice, the early treatment and the optimization of local dressings are emphasized to effectively treat the infection, biofilm formation, and excessive keratinized inactive tissues of the wound (Figure 1).

### 2.1 Surgical management: Debridement

The main management guidelines of European, Canadian and American organizations and the International Working Group on diabetes foot regard CSWD of DFU as the standard treatment method. Using sharp treatment tools such as scalpel, forceps, and spatula to remove the inactive tissue and aging and nonfunctional cells of the wound surface (Rayman et al., 2020; Schaper et al., 2020), it is not clear how much this can improve the bacterial load, but it is meaningful in removing biofilm (Wolcott et al., 2010) and controlling infection. In addition, hyperkeratosis is a special feature of diabetes-related foot ulcers, which is associated with sensory loss and chronic repetitive trauma and can also be treated by sharp debridement. Removal of hyperkeratosis is associated with a decrease in plantar pressure, which may contribute to healing (Nube et al., 2021). Patients with non-ischemic diabetes-related foot ulcers are provided with CSWD once a week, which is the main way to remove inactive tissues to promote healing. Another clinical study focuses on the treatment of diabetic foot wounds and forms a series of diagnosis and treatment processes. Doctors will meet patients at the wound care center or bedside. According to the patient’s wound condition, wound screening, debridement, wound care advice, and education will be provided gradually. Through the establishment of this effective path, the amputation rate of diabetes feet was greatly reduced. The study emphasized the necessity of early, long-term and standardized debridement (Hsu et al., 2015).

### 2.2 Intensive care: Speed dating model

It is composed of Surgeons (mainly vascular surgeons and podiatrists) and supported by endocrinologists, diabetes educators, nutritionists, infectious disease doctors, nurses, and clinical psychologists to form a rapid access rapid discipline team. It adopts a double-blind approach to conduct an overall assessment of the patient’s mental health, compliance programs, diet, and blood glucose monitoring. This method may reduce the incidence of leg amputation (Everett and Mathioudakis, 2018).

### 2.3 Adapted nutrition

The guidelines of the International Working Group on diabetes feet (IWGDF) in 2019 pointed out that “do not use interventions aimed at correcting the nutritional status of patients with diabetes feet (including supplementation of protein, vitamins and trace elements, and use of drugs to promote angiogenesis to treat diabetic foot ulcers. The aim is to improve healing rather than the best standard of care” (Basiri et al., 2020). Vitamins and minerals play an important role in wound healing, and if people lack these factors, their wound healing will be affected (Moore et al., 2020; Bechara et al., 2021). Vitamin C supplementation at the usual supplementation dose (up to 1,000 mg per day) is also considered safe because it is water-soluble and excess intake will be excreted from the urine (Bechara et al., 2021). Vitamin C supplementation is beneficial for patients with foot ulcers (Afzali et al., 2019). Vitamin D (Halschou-Jensen et al., 2021) and complex vitamin B may also be effective for the healing of foot ulcers. Both methods are relatively safe but should be performed under dietary and/or medical supervision. In addition, zinc, magnesium, omega-3, vitamin D, and probiotics have obvious benefits in wound recovery. Genistein supplementation will be a potential therapeutic nutrient that can prevent and treat delayed wound healing by regulating inflammation and oxidative stress during the inflammatory phase (Eo et al., 2016). In the treatment of complications of diabetes, oleopicroside can reverse apoptosis, regenerate tissue, restore histological tissue and reduce oxidative stress (Zheng et al., 2021). Arginine, glutamine, and β- Hydroxyl- β- Methyl butyrate may improve the healing of patients with diabetic foot ulcers (Armstrong et al., 2014). Supplementation of adult diabetes mice with standardized fermented papaya preparation (FPP) can improve skin wound healing results (Dickerson et al., 2012). These preparations may also be able to achieve rapid healing of diabetes wounds by combining with effective drug delivery systems (DDS).

### 2.4 Sterile confinement: Wound dressing

A case report of nonhealing of the wound of a patient with type 2 diabetes 7 months after cardiac surgery was carried out in Italy. In the report, the patient used the NPWT method to treat the wound for a long time after infection with MSSA, but the effect was poor. After diagnosis and treatment by the wound care team, a dressing combined with bacteria and true bacteria was selected as the healing trigger, because this dressing can interrupt the circulation of chronic or long-term inflammation. The sternal wound improved after the appropriate dressing regimen was applied. The improvement observed with sorbet was not detected in the first 7 months of NPWT (Caruso et al., 2018). Researchers believe that this wound-healing environment can greatly improve the prognosis (Castiello et al., 2019). However, at present, there are still few functional dressings applied to the human body, and the clinical transformation is difficult. In this regard, biomaterials become important players. Section 4 of this article focuses on the role of antibacterial biomaterials in wound healing.

### 2.5 Hyperbaric oxygen therapy

Systemic hyperbaric oxygen therapy (HBOT) has been proposed as a drug treatment for diabeti foot ulcers (Doctor et al., 1992). HBOT has been shown to have antibacterial effects and increase oxygenation of hypoxic wound tissues (Löndahl et al., 2010). This enhances the killing ability of neutrophils, stimulates angiogenesis, and enhances the activity of fibroblasts and collagen synthesis. In addition, hyperbaric oxygen enhances wound healing in diabetes by promoting fibroblast proliferation and endothelial cell angiogenesis (Huang et al., 2020). In the past 20 years, many clinical trials have proved that HBOT can promote the healing of diabetes wounds (Mutluoglu, 2018). HBOT is to treat patients with 100% oxygen above atmospheric pressure. This is provided either in a unit (single-person) chamber that is normally compressed with oxygen or in a multi-position chamber (multi-person) that is compressed with air, where oxygen is delivered by a mask or mask. Bring the effect of improving leukocyte function, improving ischemia-reperfusion injury, and neovascularization due to the increase of local growth factors and the release of autologous progenitor cells (Thom, 2011). However, hyperbaric oxygen also has its unavoidable harm. Including various forms of barotrauma, central nervous system (CNS), and pulmonary oxygen toxicity, as well as ocular side effects, and claustrophobia (Thom, 2011; Heyboer et al., 2017). In response, researchers have introduced dressings rich in oxygen or other effective gases to inhibit the growth of anaerobic bacteria or provide more positive effects on the tissue. This effective effect does not need to be mobilized and may be achieved only by topical dressings or stents.

### 2.6 Skin tissue engineering

It may be difficult to achieve the healing of the wound by simply supplementary treatment. The development of tissue engineering, bioengineered skin substitutes, and genetic growth factors have made great progress in the treatment of chronic skin ulcers in recent years (Morimoto et al., 2015). At present, there are few reports of skin tissue engineering materials applied in the clinic, but it must be said that this will be the inevitable trend in wound repair (Gholian et al., 2022). Bioengineered skin substitutes for DW include amniotic membrane, autologous stem cell therapy, fibroblast-derived dermis, and porcine small intestinal submucosa (PSIs). The biological activity of PSIs includes GF, such as TGF-β, vascular endothelial growth factor (VEGF), and FGF, which limit the destructive movement of MMPs and promote angiogenesis to help neovascular development (Tallapaneni et al., 2021).

Some studies used bioengineered skin for control experiments, including 880 subjects. Bioengineered skin (BS) has significant advantages in effectiveness and safety, and the risk of infection is significantly reduced (Teng et al., 2010). Another study used degradable gelatin dressings (applied as DDS of bFGF in clinical trials) to load PrP to treat diabetic wounds. A total of 30 patients were included in the study. This combination therapy may be an alternative to bioengineered skin substitutes containing live cells and lead to substantial progress in chronic skin wound management (Morimoto et al., 2015). Collagen dressings can serve as skin substitutes for natural extracellular matrix (ECM) to guide the complex cellular interactions necessary to promote the migration of keratinocytes and fibroblasts (Amirrah et al., 2020). The engineered skin grafts with matrix blood vessel cells encapsulated by fibrin collagen hydrogel also have good effects on the wounds of patients with diabetes. It is hoped that the grafts can be truly applied to the clinic through multi-center clinical trials. Among them, scaffolds composed of a combination of type I collagen and fibrin can improve mechanical properties and enhance the ability of SVF microvascular formation (Nilforoushzadeh et al., 2020). Next, the treatment method incorporating stem cells has also become a new research hotspot. Placenta-derived mesenchymal stem cells were isolated from human donor placentas and cultured in electrospun gelatin nanofiber scaffolds (GNs). The results showed that the implantation of HPD-MSCs in GNS could accelerate wound healing of DFU patients (Meamar et al., 2021). The gradual promotion of clinical practice can better promote the development of basic research, and clinical application is also the ultimate goal. Therefore, it is necessary to provide more directions for clinical trials through multi-faceted biocompatibility verification. The following summary will focus on the application of biodegradable materials in diabetes wounds through the addition of various factors based on biodegradable materials.

It is worth noting that, during the wound healing process, the dressing protects the injury and contributes to the recovery of dermal and epidermal tissues. As per the sophisticated definition of tissue engineering described at a National Science Foundation workshop, scaffolds are the best materials for restoring, maintaining, and improving tissue function (Chaudhari et al., 2016). In other words, dressings are more likely to provide a conducive environment for healing, while scaffolds are more likely to serve as a connection and storage of active ingredients.

## 3 Biodegradable biomaterials

The targeted delivery of drugs has been a focus topic in recent years. During systemic administration, the ineffective vasculature tissue of the wound can effectively deliver drugs, and the half-life of the drug itself and the unpredictable wound environment make it difficult for the drug to act accurately (Wang et al., 2019). Therefore, for the treatment of diabetes wounds, it seems to be more inclined to local action. Although there is still great controversy about carrying antibacterial drugs for local action, although it can reduce the dosage, it is difficult to ignore the impact on the local microenvironment (Bhise et al., 2011).

As an ideal dressing, it should have the following characteristics (Vuerstaek et al., 2006; Chong et al., 2007; Boateng et al., 2008; Das and Baker, 2016) for diabetes wound:

• Liquid balance: it can not only absorb excessive wound exudation but also maintain the moist environment of the wound;
• Avoid further wound damage;
• Prevention and control of bacteria present or colonization;
• Eliminate dead space;
• Debridement of necrotic tissue;
• Does not affect the activity of surrounding tissues;
• Do not cause an allergic reaction or shed substances that can cause foreign body reaction;
• Convenient replacement, no pain, low price;
• Transparent, easy to observe and monitor the wound condition;
• The tensile strength is in the range of 0.7–18 mPa (Samadian et al., 2020);
• It can contain endogenous cells or active factors and promote cell proliferation, differentiation, and migration, to promote wound healing (Chereddy et al., 2016).

Combined with the above theoretical support, researchers are committed to developing biomaterials closer to ideal dressings for wound repair in diabetes patients. The development of different materials also provides us with more alternative directions.

### 3.1 Classification and characteristics of degradable biomaterials

Biodegradable biomaterials can be mainly divided into two categories: natural polymers and synthetic polymers. Natural polymers are easy to obtain and have strong biocompatibility, but their physical and chemical properties are not controlled and their types are limited (Zhang et al., 2021; Zhang et al., 2022). However, synthetic polymers, due to their high controllability, effectively make up for this defect of natural materials. We summarized the table for specific material classification and characteristics of each material (Table 1). However, no matter what kind of material, its characteristics are mostly reflected in its good basis as a wound dressing, such as degradability, biocompatibility, and mechanical properties. However, the disadvantages of various basic materials used for wound healing are also obvious. For example, synthetic polymers lack the ability of cell recognition, so it is less likely to be used alone (Vogt et al., 2021). Therefore, to vantage the advantages and avoid the disadvantages of materials, researchers hope to mix materials using materials to achieve the optimization of materials.

**TABLE 1:** Classification and characteristics of biomaterials.

Source	Classification	Ingredient	Preparation procedure	Advantages	Disadvantages	References
Natural polymers	protein-based	collagen	Hydrogel, nanofiber scaffold, sponge, etc	Easy to get, excellent biocompatibility	Suitable for wounds with less exudation; Contraindicated for patients with third-degree burns and allergic patients	Tang et al. (2018)
		silk fibroin	3D scaffolds, nanofibers, films, etc	biocompatibility, biodegradability, flexibility, adherence, absorption of exudates, minimal inflammatory reaction	Degradation is slow and may cause sensitization	Kundu et al. (2013)
		gelatin	films, sponge	excellent biocompatibility	Do not apply to the wound with more exudation	Xiao et al. (2021)
	polysaccharides	agarose	films	Water expansion capacity (4–5 times) and tensile strength (30–50 MPa)	Poor biomechanical properties	Taghipour et al. (2020)
		alginate	Film, sponge, hydrogel, etc	Stop the bleeding. Water absorption; Antibacterial; Can be combined with laser therapy	It is porous and non-adhesive. It needs to be protected and secured with secondary dressing	Taghipour et al. (2020)
		hyaluronic acid	Hydrogels, scaffolds, etc	No scar healing, good angiogenesis effect, nerve repair effect, can be modified to simulate the effect of ECM	Poor adhesion, poor stability *in vivo*	Kim et al. (2021)
		chitosan	Bandages, ointments, films, sponges, hydrogels, etc	Hemostatic, antibacterial, simple method of blending with other effective substances	Contraindicated with strong oxidants	Muxika et al. (2017)
Syntheitc polymers	polyesters	PLA	Scaffolds, nanoparticles, etc	Strong mechanical properties, strong absorbability, good air permeability	Acidic products may cause local foreign body reactions	Hajikhani et al. (2021)
		PGA	Thin films, nanofiber scaffolds	Hydrophilic, biocompatible, biodegradable and non-toxic	It has no tissue adhesion ability or wound healing effect, and is often used in combination with fibrin glue	Zha et al. (2022), Kouketsu et al. (2021)
		PLGA	Nanofibers, membranes, microspheres, hydrogels, nanoparticles	Easy degradation, possibility of adjusting surface and physicochemical properties, continuous drug release	Low drug loading and high material strength may induce local irritant reaction	Chereddy et al. (2016)
		PHB	Nanofibers, nanoparticles, scaffolds	Good mechanical properties, can be degraded in the ideal time range, insoluble in water	Poor antibacterial effect	Sanhueza et al. (2021), Avossa et al. (2021)
		PCL	Nanofibers, nanoparticles, scaffolds	Poly (ε-caprolactone) (PCL) is biocompatible and biodegradable polymer which can be electrospun easily at low voltages and is able to provide required scaffold mechanical resistance in aqueous environments.The cell affinity, stability and strength of nanofibers were improved	Higher processing conditions; It is not effective on its own	Croisier et al. (2014)
	Polyanhydrides	poly (sebacic acid)	Hydrogels, scaffolds, etc	Stable batch to batch consistency; Easy to customize	Lack of cell recognition; Difficult to apply independently	Vogt et al. (2021)
		polyamino acids	Nanofibers, hydrogels, etc	Strong biocompatibility, bacteriostatic, hemostasis, drug delivery, good film forming effect	The preparation process is demanding and the synthesis is difficult	Shen et al. (2021), Ji et al. (2022)
	phosphorous based polymers	polyphosphates	Hydrogel, hydrocolloid, etc	Hemostasis, providing energy for cell proliferation, and effectively enriched in the wound site	The preparation process is demanding and the synthesis is difficult	Al-Musawi et al. (2020)
	Others	polyurethane	Foam, hydrogel, film, etc	It has adjustable hard and soft chain segments and modifiable chain extenders	The biocompatibility is relatively poor	Lutzke et al. (2016)

First of all, composite materials themselves can guide wound healing. Various cells and active factors involved in the process of wound healing are affected by physical signals, mechanical signals, chemical signals, inorganic signals, and other ways of the materials themselves, thus changing the behavior of cells (Castaño et al., 2018). In other words, materials mainly interfere with the internal factors of the wound itself.

After that, the ability of the material itself entered a “threshold” state, and the effect of material processing and changes on wound healing was not significantly improved. Combined with the basic principle of wound healing, researchers focused on external factors that may be involved in wound healing. For example, by improving the properties of the materials or the proportions of various components of the composite materials, we can find the drug delivery system (DDS) most suitable for carrying exogenous drugs, or by innovating the processing technology of the materials, we can improve the internal structure of the materials, to achieve greater cell adhesion.

However, the exertion of the characteristics of the materials themselves is still the most serious issue. Through the literature summary, we summarized and sorted out the second table, which sorted out the combination methods, main functions, and mechanisms of composite materials after combination (Table 2). The combination of natural materials and synthetic materials needs to consider the synthesis efficiency of natural materials and the maintenance of physical and chemical properties after synthesis. Although the mixing of multiple synthetic materials is controllable, most of the required manufacturing processes are complex, and it is difficult to ensure that the biological compatibility after synthesis can still maintain the efficacy of a single polymer.

**TABLE 2:** Composite material combination and wound healing mechanism.

Complex method	Ingredient	Function	Mechanism	References
Natural-Natural	Col-CS	hemostasis	The binding affinity of CS was increased	Tripathi et al. (2021)
	Col-SF	Moisturizing; The biodegradability is controllable; The synergistic effect on cell viability was improved	inflammation stage: ↑IL-6, IL-1β, TGF-β1, TGF-βR2↑; MSC + Col + SF→Blood vessels mature↑, Cell adhesion (>60%)	Naomi et al. (2020)
	CS-Col-alginate (CCA)	Good water absorption; Good mechanical properties; Good cell compatibility; The seawater intrusion can be prevented for 4 h	EGF, bFGF, TGF-β and CD31↑	Xie et al. (2018)
	Col-CS + IGF1	The 3D structure has a large number of pores, which is conducive to cell adhesion, proliferation, and liquid exchange	Erk1/2 mediates IGF-1-induced proliferation and migration of BMSC	Xia Y. et al. (2020)
	Gel-cellulose-bFGF	Improved biocompatibility (Gel and bFGF were fixed by hydrogen bond)	Composite material specific ↓ Mir-29b-3p level, targeting Akt/GSK-3β/β-catenin pathway to regulate the biological function of endothelial cells	Yu et al. (2020)
		Oxygen was permeated and water vapor was controlled to evaporate and absorb wound exudate		
	CS-alginate-Velvet antler blood peptide	Heat sensitivity, antioxidant properties, antibacterial properties, safety, sustained drug release, and the dressing locks water and provides oxygen to the wound	PI3K/Akt/mTOR pathway SIRT1/NF-kB pathway	Hao et al. (2022)
Natural-Synthetic	PCL-Col-ZnO-VEGF fibrous	Antibacterial, angiogenesis, cell proliferation	TGF-β, CD31 ↑	Li P. et al. (2021)
	PHB-Gel-OSA-Col	OSA: osthole amine - for antibacterial	promote the regeneration of dermal papilla layer and hair follicle	Kandhasamy et al. (2017)
		Composite material is stable to enzymatic degradation		
	nCur-PVA-Col	Cytotoxicity ↓; Fibroblast adhesion and proliferation ↑	During the stages of skin repair and remodeling, Tgf-β ↑, collagen maturates	Leng et al. (2020)
	PVA/Alginate hydrogel	Moisturizing, anti oxidation and slow release	regulation PI3K/Akt pathway	Chen G. et al. (2020)
	PCL-HA Nanofiber scaffold	Significantly enhanced cell infiltration	It is affected by the changes of cell adhesion receptor-integrin β1 and the formation and distribution of neotins	Qian et al. (2015)
			Tgf-β/MMP-2 pathway for cell motility	
	VMT-PCL	Promoting angiogenesis	HIF-1α pathway	Huang et al. (2022)
			VEGF,SDF-1α,eNOs↑	
			Col III, FN, bFGF↑(PCL/5%VMT)	
			M2↑	
	PCL-CA-CSThree-layer nanoscaffolds	Mimics the extracellular matrix	PCL/CA→YAP→Notch pathway	Lin et al. (2021)
			Proteomics of differential expression of ribosome-related proteins and metabolic pathway-related proteins	
	PCL-Gel Nanofiber scaffold	Promote the adhesion, proliferation and migration of human keratinocytes (HaCaTs) and HUVECs; Promote angiogenesis, collagen deposition, re-epithelialization	Activating epithelial-mesenchymal transition (EMT) and endothelial-mesenchymal transition (EndMT)	Lv et al. (2017)
	SF-PCL	The ability to regulate macrophage polarization	The ability of noncovalent binding to IL-4 to regulate macrophage polarization	Qian et al. (2018)
			The effect of promoting macrophage polarization in the early stage can significantly inhibit the degree of late foreign body reaction	
			MARK, PI3K, JKN and Nf-Kb pathways regulate macrophage polarization	
Synthetic-Synthetic	HAP-ZrO2-GO-PLA	Rough surface: cell adhesion ↑	Go surface coordination keeps oxygen-containing groups that interact with the surrounding environment highly active	Al-Wafi et al. (2021)
	PHBV-HMK nanofibers	HMK has strong biocompatibility	HMK promoted cell proliferation	Ye et al. (2020)
		PHBV has strong mechanical strength and antibacterial property		

### 3.2 Development and changes of degradable biomaterials

Biodegradable biomaterials have gradually changed from the initial covering to the cell culture matrix with bionic function. Biomaterials can not only become scaffolds or dressings for wound healing but also create a favorable environment for cell growth. This change has promoted the progress of materials science.

#### 3.2.1 Temporary wound cover

The original intention of designing the wound dressing was simply to cover the wound with a shielding “cloth” instinctively for protection of the wound Gauze can absorb exudation, keep the environment moist, and can be made into sterilized products by simple methods, but its shielding ability is poor, and dressing change is often accompanied by pain or secondary damage (Jones, 2006). Given this situation, a transparent elastic dressing made of polyurethane was derived to shield bacteria and allow gas exchange, but it cannot be absorbed when too many exudates. Foam dressings seem to perfectly solve this problem, but excessive water absorption is difficult to achieve drug delivery (Vermeulen et al., 2005). As a cross-linked three-dimensional network structure, the hydrogel has variable morphology and adjustable swelling. However, its permeability to gas limits its use on infectious wounds. Therefore, it is unwise to use hydrogel dressing alone for diabetes wounds (Annabi et al., 2014). The hydrogel made of alginate has both antibacterial and water absorption. Hydrogel dressings made of gelatin, pectin, or hydroxymethyl cellulose seem to have a similar effect. The dressings at this stage are mostly used to supplement or provide temporary solutions after problems occur according to clinical needs.

#### 3.2.2 Regenerated scaffold or environment

No matter how comprehensive the cover is, it can not replace the lost tissue. Therefore, new biomaterials will focus on the establishment of bioactive scaffolds for the wound. Fiber scaffolds affect cell arrangement, shape, and function by mimicking ECM fiber organization. The main form of simulated ECM is a hydrogel, which shows good thermal stability, controllable biodegradation, good swelling, and smooth surface morphology (Thangavel et al., 2017). *In vivo* wound closure examination using STZ-induced mice showed that the full-layer wound wrapped with a chitosan sponge containing TMC nanoparticles healed faster than that of ordinary chitosan wound dressings. The biological activity of TMC nanoparticles is a good antibacterial effect, thus improving the wound healing of diabetes injury (Xia G. et al., 2020). These bioactive scaffolds or the enclosed environment formed between the wound and the dressing can solve the problem that temporary ECM is difficult to generate due to the presence of too many MMPs (Griffin et al., 2015).

#### 3.2.3 Restore the natural structure of ECM

As mentioned above, collagen and hyaluronic acid are components of natural ECM and have good histocompatibility and biodegradability *in vivo*. Studies have shown that scaffolds bound to extracellular matrix (ECM) proteins can regulate cell behavior and improve wound healing. However, most brackets that contain the ECM cannot capture the dynamic functions of the ECM. The collagen fiber structure in ECM can be simulated by electrospinning technology (Kim et al., 2017). Nanofiber scaffolds mimic the compositional transition of ECM during wound healing and may have great potential to promote skin regeneration through dynamic regulation of the microenvironment (Zhao et al., 2016; Sun et al., 2021).

The dressing developed according to this idea has good cell compatibility with keratinocytes and fibroblasts and enhances their cell proliferation and migration ability *in vitro*. The supportive nanoscale matrix mimicking ECM promotes increased collagen deposition in the wound bed, thereby accelerating the complete healing process through massive tissue regeneration and functional recovery (Nanditha and Kumar, 2022). Through the electrospinning method, researchers have manufactured polylactic acid glycolic acid/collagen nanoscale mats, and functionalized the surface with wound healing peptides, loaded chitosan nanoparticles and micron-sized particles to form an extracellular matrix (ECM) - like structure with bionic functions (Yin et al., 2020). The researchers also found that iPSCs-derived fibroblasts (“post IPSF”) can promote angiogenesis by producing more ECM than IPSF precursor cells (somatic precursor - “IPSF precursor”), and their ECM has the characteristics of fetal ECM. This initial state of ECM recovery, with higher cell content, higher vascular endothelial growth factor (VEGF), and higher interleukin-1 receptor antagonist (IL-1ra) (Santarella et al., 2020).

#### 3.2.4 Promote cell proliferation, differentiation, and migration

The pore diameter of the sponge material can be from 50 μ M to mm, which is conducive to cell infiltration, migration, and signal transduction (Katoh et al., 2004). The results showed that the sponge scaffolds made of collagen could promote the adhesion, migration, and proliferation of fibroblasts and keratinocytes cultured on its surface. However, the collagen from lactating animals is degraded rapidly and may also cause transgenic diseases (Chandika et al., 2015; Ramanathan et al., 2017). Adding glycosaminoglycan (GAG) to the hydrogel can also promote cell infiltration and proliferation (Kirker et al., 2002). Surface plasma treatment can improve the hydrophilicity of materials, thus playing a similar role (Chandrasekaran et al., 2011). Studies have shown that hFDSPC-CM can pass through TGF- β/Smad signaling pathway promotes the proliferation and migration of cells (keratinocytes and fibroblasts) in the wound surface of diabetic mice and realizes the wound healing of diabetes mice by combining with HA (Xin et al., 2021).

#### 3.2.5 Promote angiogenesis

Although collagen is a widely used wound healing material, its angiogenic ability is poor and its explanation speed is fast. Therefore, fibrin-based hydrogels were designed to effectively promote angiogenesis and cell recruitment (Zeng et al., 2015; Chung et al., 2016). Or electrospinning using these natural proteins to improve their angiogenic ability (Xu et al., 2019). Recent studies have found that a 3D short fiber sponge provides an oxygen-rich environment for cell growth, which is conducive to the 3D proliferation and growth of HUVECs, stimulates the expression of VEGF, and well promotes the angiogenesis of HUVECs (Li Y. et al., 2021). But the promotion of angiogenesis and cell function may be concomitant.

#### 3.2.6 Sterilization characteristics

**FIGURE 2:**
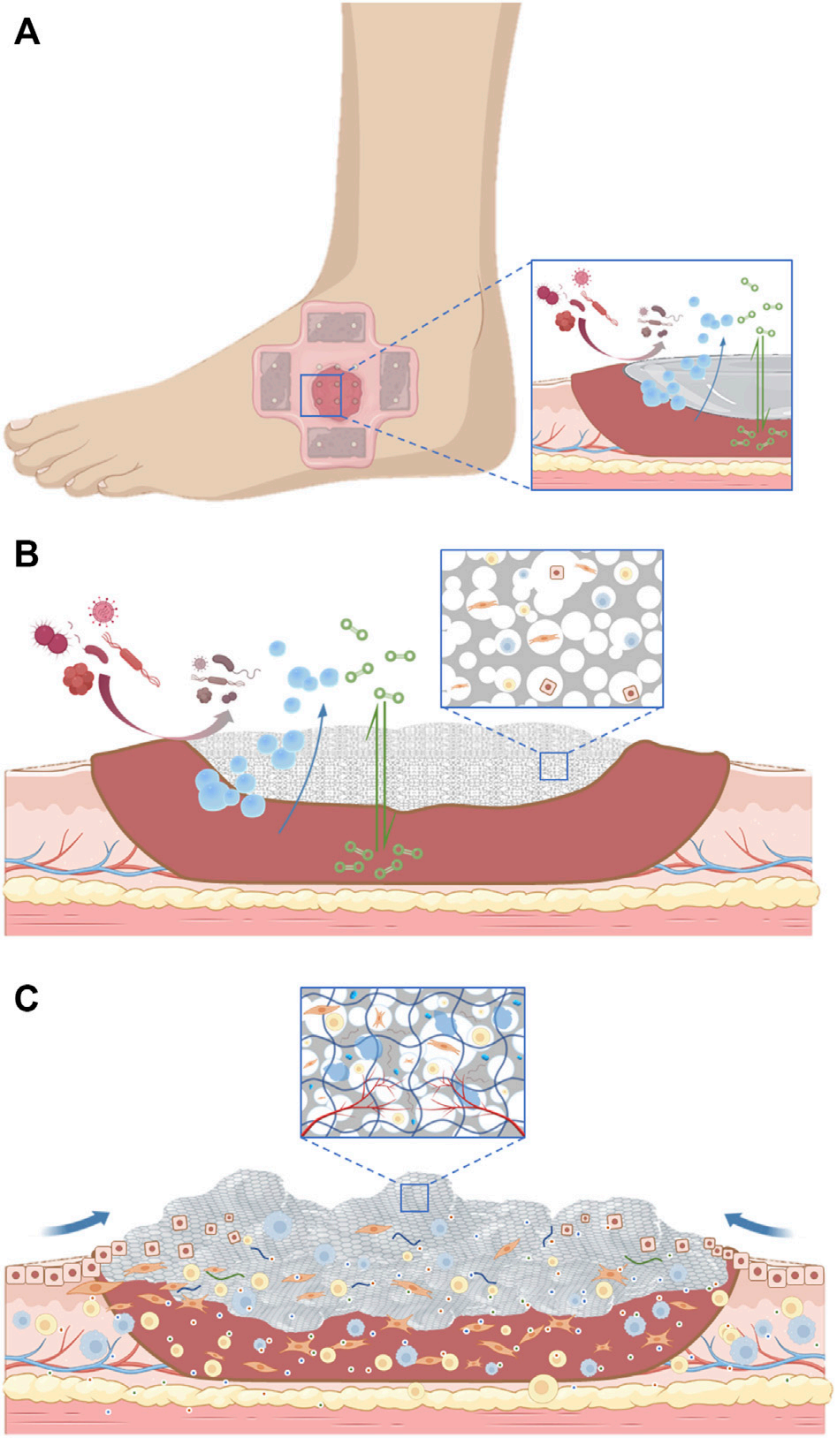
**(A)**. The initial action of the dressing applied to the wound. **(B)**. The dressing serves as a scaffold for tissue regeneration. **(C)**. Dressings are gradually becoming biomimetic.

One of the main characteristics of a diabetes wound is the complex infection of the wound. Chitosan has unique advantages in dealing with this problem. It has been found that chitosan not only promotes cell adhesion and migration but also has a bactericidal ability (Miguel et al., 2014). The concentration of 188 g/ml enables the hydrogel given to chitosan to inhibit the growth of bacteria, and the electrospun chitosan material has a similar effect. The collagen sponge with anti-infective bioactive molecules also effectively restored the normal function of fibroblasts and keratinocytes in infectious wounds (Ramanathan et al., 2017). The latest research shows that glycopeptide hydrogel accelerates the reconstruction of full-thickness diabetes and scalded skin infected by methicillin-resistant *Staphylococcus aureus* (MRSA) by coordinating a large number of M2-type macrophages, reducing inflammation and promoting angiogenesis (Liu W. et al., 2022). We summarize the evolution of wound dressing function through a set of figures (Figure 2).

### 3.3 Flexible applications of biodegradable biomaterials

After improving the composite and modification of biomaterials themselves, we hope that biomaterials can realize the exogenous supplement of missing active ingredients, at the same time, maximize the activity of endogenous cells and growth factors, and on this basis, carry some drugs or inorganic substances, to realize the flexible application of biomaterials based wound healing materials. As mentioned in the second part, there are some extremely effective methods in clinical application, but they are accompanied by serious problems, such as systemic adverse reactions of hyperbaric oxygen chamber therapy or the uniformity of wound dressing. The flexible application of biodegradable biomaterials provides a supplement to this shortcoming in clinical practice. Therefore, according to the characteristics of diabetic wounds, we expect biomaterials to be antibacterial, anti-inflammatory, pro-angiogenic, and restore appropriate environmental conditions for wound healing.

#### 3.3.1 Purpose of active ingredient accumulation

The three elements of tissue repair and reconstruction include scaffolds, cells, and growth factors. Whether it is the repair of bone defects (Ren S. et al., 2022) or the healing of skin wounds, these three factors are inseparable. Therefore, based on degradable biomaterials, adding cells and growth factors can meet the environment and requirements of tissue regeneration to the greatest extent.

In addition, in a large number of studies, degradable biomaterials loaded different drugs to achieve targeted drug delivery. In addition to the improvement of medication, it is more important to promote some drugs with short half-lives to protect their activity *in vivo*. In addition, the presence of a large number of proteases in the microenvironment of diabetes wounds leads to the degradation of GFS, which further hinders angiogenesis and diabetes wound healing. Some studies have shown that ECM simulation and immune regulation can be achieved through the modification of the material itself (Liu W. et al., 2022), to achieve the antibacterial effect. However, the healing of diabetes wounds does not depend solely on the antibacterial effect.

Admittedly, what we need to prove is that each accumulated component has a synergistic effect, not an opposite effect. The cytotoxicity of chemosynthetic drugs may affect the activity of cells and cell components, while the preparation process and mode of action of scaffold materials may not necessarily reach the ideal state. However, by observing the accumulation of time, environmental assessment at different stages, and the changes in the microenvironment *in vivo*, we hope to truly achieve the most ideal environment for wound healing, and even make use of some specific environments, such as diabetes, to respond to pH or temperature, to accumulate effective ingredients and make adjustable personalized wound healing materials.

#### 3.3.2 Functional enhancement of biodegradable biomaterials

**FIGURE 3:**
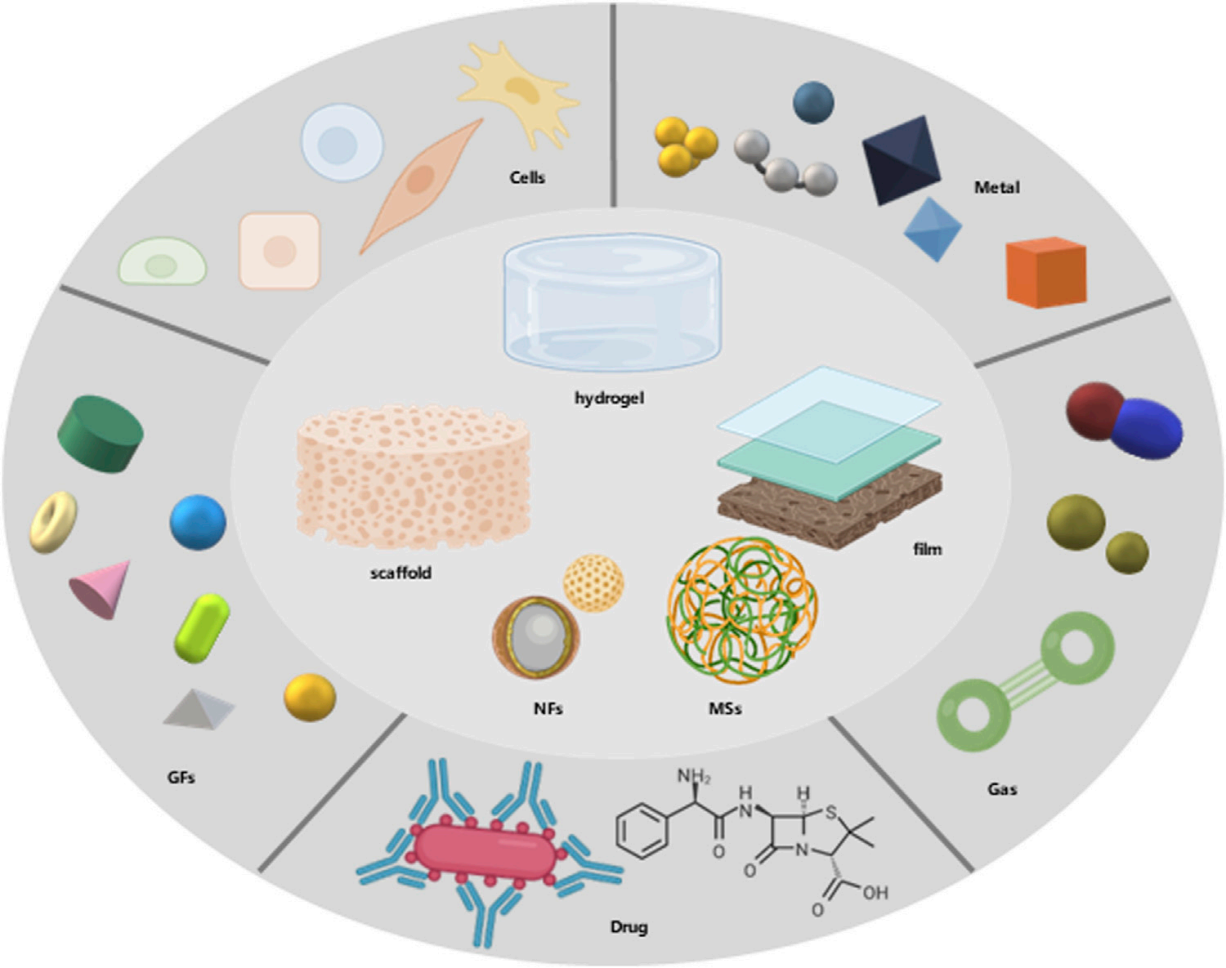
The combination of composite biodegradable biomaterials.

The application research of degradable biomaterials is always inseparable from material-based property modification. Of course, new basic materials are emerging constantly. The current research focus is to combine materials with various drugs, cells or ions, and other components that have positive effects on wound healing to simulate the optimal and ideal conditions for wound healing (Figure 3). As mentioned above, we should focus on the functional improvement of wound dressings and skin tissue engineering. The local oxygen-rich environment and drug-loading conditions are particularly important for functional improvement.

##### 3.3.2.1 Loading drugs

The most important means to combat chronic wounds related to diabetes is to use drugs for intervention. Through the mastery and application of the properties of biomaterials, various drugs can be carried and released, form a drug delivery system (DDS) based on degradable biomaterials, and make the pharmacological effect play a better level. However, the research in recent years tends to apply more known drugs in other fields, and develop more combinations of materials through the modification of natural extracts. Because biomaterials themselves can achieve the partial antibacterial effect through modification, it seems that the local antibacterial drug carrier is conservative for the antibacterial effect of wounds (Garin et al., 2021; Zhou et al., 2022).

###### 3.3.2.1.1 Anti-diabetes drugs

PLGA and metformin were dissolved in 1,1,1,3,3,3-hexafluoro-2-propanol (HFIP) and spun into nanofiber membranes by electrospinning. The metformin in PLGA could be slowly and persistently released for more than 3 weeks due to the stable degradation performance of PLGA, thus promoting the healing of diabetes wounds (Lee et al., 2014). Similarly, to achieve the sustained release of metformin, cam et al. Manufactured bacterial cellulose gelatin nanofibers co-loaded with glibenclamide and metformin (Alven et al., 2022). Core-shell nanofiber bioactive insulin-loaded PLGA scaffolds can also be used to repair wounds in diabetic rats. Mechanical analysis of the insulin-loaded nanofibers showed that the elongation at break was 164.3% ± 27.2%, and the tensile strength was about 2.87 ± 0.07 MPa, which was similar to human natural skin. PLGA and insulin obtained scaffolds by coaxial electrospinning, and insulin was continuously released for 4 weeks. Nanofiber core-shell insulin-loaded scaffolds reduced the content of type I collagen *in vitro* and increased the growth factor in vivo-β, which can prolong the release of insulin and promote the healing of diabetes wounds (Lee et al., 2020b). The pioglitazone-loaded PVP-PCL nanofiber pad showed an initial rapid release of the drug in type I diabetes mice, followed by a sustained release mode. The sustained release of pioglitazone and its good cytocompatibility led to an accelerated wound-healing process (Cam et al., 2020). *In vitro*, the lira (Yu et al., 2020) reversed the inhibitory effect of high glucose (Hg)-induced endothelial cells on proliferation, migration, tube differentiation, and VEGF secretion. In the mechanism study, the lira was found to specifically reduce the level of mir-29b-3p and target Akt/GSK-3 β/β- The catenin pathway regulates the biological function of endothelial cells.

###### 3.3.2.1.2 Antibiotics

Continuous topical use of antibiotics can avoid wound infection and related bacterial colonization. Therefore, the method of adding antibiotics to local dressings and making them sustained release has a positive effect on wound healing (Lee et al., 2020a). Local use of DCH to treat wound infection is a common clinical method. Because the local concentration and treatment cycle are difficult to control, the side effects are large in the clinical application process. DCH-encapsulated polylactide (DCH/PLA) nanofibers were prepared by electrospinning. Among them, the rate of DCH release can be controlled within 3d-2w, which can specifically fight against E. coli and S. aureus, thus promoting wound healing (Cui et al., 2019). Gentamicin-loaded collagen sponges also have similar effects (Lipsky et al., 2012). Vancomycin-loaded N-trimethyl chitosan nanoparticles (VCM/TMC NPs), as a potential drug delivery system, have high intracellular penetration and effective intracellular antibacterial activity (Fukushima 2016; Zhang Y. et al., 2017). Ahmed et al. (2018) reported that calcium alginate-ba lyophilized sheets combined with ciprofloxacin were used for wound management of diabetes patients with microbial infection to inhibit and prevent reinfection caused by Gram-positive and Gram-negative bacteria. Loading the antibiotic ciprofloxacin into the wafer can be used to treat diabetes injury caused by bacterial infection, and it shows good cytocompatibility with human keratinocytes. Another hydrogel loaded with fusidic acid has excellent flexibility and elasticity based on rapid film formation, and produces high drug release. The FFH loaded with sodium fusidate with the weight ratio of sodium fusidate/PVP/PVA/propylene glycol/ethanol/water of 1/2/12/3/8/74 can rapidly form a corresponding dry film at the wound site and can be stable at 45°C for 6 months (Kim et al., 2015). There are also studies using PVA foam-loaded methylene blue and gentian violet for anti-local infection treatment (Coutts et al., 2014). In the future, we should implement appropriate local dressings according to the results of bacterial culture and drug sensitivity tests of the wound.

###### 3.3.2.1.3 New trials of drugs for the treatment of other diseases

PLLA basedoaded electrospun fiber membrane, which is coated with dimethylglyoglycine-loaded mesoporous silica nanoparticles, promotes the proliferation, migration, and angiogenesis-related gene expression of human umbilical vein endothelial cells (Ren et al., 2018).

Through electrostatic interaction, chitosan, heparin, and poly (γ- Glutamic acid). Hydrogels have a good swelling ability and show typical viscoelasticity and good mechanical properties in rheological tests. The fibroblast proliferation test showed that the hydrogel had cell compatibility. Its ability to promote healing is attributed to the wound healing function of chitosan loading superoxide dismutase by promoting cell proliferation and by reducing ROS production in the wound bed (Zhang L. et al., 2018).

Neurotensin (NT), also known as neurotensin, is named because it has an obvious antihypertensive effect and exists in nerve tissue. It is easy to separate from other peptide hormones due to its specific vascular effect and blood pressure lowering effect. The main problem of the neuropeptide problem is the short half-life and low bioactive concentration in the peptide-rich wound environment. Moura et al. (2014) reported the application of chitosan-based foam loaded with neurotensin in wound healing of diabetes. This material can increase the migration of fibroblasts and the expression and deposition of collagen (COL1A1, COL1A2, and COL3A1), to promote wound healing. The *in vitro* drug release curve of neurotensin-loaded PLGA cellulose nanocrystalline nanofiber membrane indicates that neurotensin is continuously released from the nanofiber membrane and the full-thickness wound healing of diabetes rats is faster (Zheng et al., 2018).

Ginkgolide B (GB), a natural product extracted from Ginkgo biloba leaves, has been used to treat cerebrovascular and cardiovascular diseases, mainly due to its antioxidant, anti-inflammatory, and proliferative effects. Through the combination of high molecular weight hyaluronic acid and ginkgolide B, anti-inflammatory and angiogenesis can be achieved. However, at present, only the comparison with ordinary dressings has been achieved, and its effectiveness cannot be evaluated (Wang L. et al., 2022).

Diosmin is a drug for enhancing venous tension and a vasoprotective agent. For the lymphatic system, it can increase the speed of lymphatic drainage and the contraction of lymphatic vessels, improve lymphatic reflux and reduce edema. A composite wafer was obtained using sodium alginate: gelatin with 1.5%/1.5% w/w, and Diosmin was continuously released within 8 h. Complete epithelial regeneration, well-organized dermis, well-formed granulation tissue, and mature collagen bundles were observed in the treated rats, and the stability of diosmin nanocrystals was maintained at the same time (Atia et al., 2019).

Hyaluronic acid (HA)/poly (lactic acid co glycolic acid, PLGA) core/shell fiber matrix loaded epigallocatechin-3-O-gallate (EGCG) (HA/PLGA) was fabricated by coaxial electrospinning. HA/PLGA-E core/fiber matrix is composed of randomly oriented submicron fibers with a 3D porous network structure. EGCG was uniformly dispersed in the shell and sustained uniformly for 4 weeks. In streptozotocin-induced diabetes rats, this novel composite enhanced re-epithelialization/neovascularization and increased collagen deposition (Shin et al., 2016).

##### 3.3.2.2 Loading cells

###### 3.3.2.2.1 Provide appropriate ECM for stem cells

The microenvironment change induced by diabetes hurts the wound repair function of mesenchymal stem cells. Therefore, there are studies to counteract the influence caused by the microenvironment of diabetes by increasing photobiological regulation (PBM) and enabling mesenchymal stem cells to play a repair role (Fridoni et al., 2019). PBM can synergize with ADSC to jointly regulate the inflammatory response, increase wound strength and wound closure rate, and significantly reduce CFU (Ebrahimpour-Malekshah et al., 2020).

Lipolysis of dermal adipocytes contributes to wound healing by regulating inflammatory macrophage infiltration. In addition to mobilizing lipid reserves, the wound environment also induces changes in the plasticity of adipocyte cells, making adipocytes at the edge of the wound become wound bed myofibroblasts that generate ECM during the proliferation phase of repair (Shook et al., 2020).

The initial attempt was to collect allogeneic adipose stem cells (ASCs) from the inguinal fat of normal rats, create ASC sheets using cell sheet technology, and transplant them to the full-thickness skin defect of diabetes obese rats. A variety of angiogenic growth factors (VEGF, HGF, TGF- β 1. IGF-I, EGF, and KGF) accelerate wound healing by promoting angiogenesis (Kato et al., 2015).

However, it is difficult to obtain and prepare large-scale stem cell sheets alone. Due to the repulsive force between cells and the binding force between cells and the substrate, it is difficult for stem cells on the wound surface to act effectively. By using soluble cell adhesion molecule (CAM) to engineer and transform cells, a powerful cell sheet is formed, which makes it possible to obtain a unique method of large-scale hADSC sheet (Na et al., 2018). This kind of stem cell sheet made by the photothermal principle also provides a new idea for the combination of materials and stem cells.

Oxygen tension is an important regulator of stem cells, controlling their homing and implantation in injured tissues. The underlying mechanism is the transient increase of reactive oxygen species/nitrogen species based on HBOT. Although a temporary increase in oxidative stress at the wound site may have deleterious effects, these active substances act as important signaling molecules to activate hypoxia-inducible factor (HIF) -1 when oxygen levels fall to normal levels. Subsequently, VEGF is activated, thereby recruiting endothelial progenitor cells and activating important antioxidant defense. The obtained results showed that the combination of MSC with HBOT improved collagen synthesis and increased neovascularization and epithelialization in the wound bed, supporting its therapeutic application (Peña-Villalobos et al., 2018).

The sprayable gel hydrogel is easy form a thick film on the wound surface after being crosslinked by blue light. The simulated neutrophil nanoparticles composed of GOx and CPO dual enzyme systems are encapsulated in zif-8 nanoparticles, which can effectively reduce the glucose concentration around the wound of diabetes and produce HClO to inhibit bacterial growth. In addition, the generated HClO plays a role in inhibiting scar formation to ensure efficient treatment of diabetes wounds, the wounds almost recover, and no scar formation on day 21 (Liu C. et al., 2022).

###### 3.3.2.2.2 Promote the function of stem cells

Mesenchymal stem cells stimulate cell migration, neovascularization, epithelial regeneration, and new wound bed formation and maturation (Kuo et al., 2011). In addition, they can also reduce the inflammatory response, enhance wound contraction, and can improve healing (Hu et al., 2018). The excised wounds of diabetes treated with HUC MSCs showed enhanced microvessel density and vascular synthesis ability, which were caused by IGF and TGF- β Upregulation (Moon et al., 2017). TGF- β It plays an important role in the whole wound healing process, mainly by recruiting inflammatory cells to the wound area from the early stage until the late stage of epithelial regeneration, involving the production and remodeling of ECM and the migration and differentiation of fibroblasts and keratinocytes to influence the proliferation stage (Pakyari et al., 2013).

Polyethersulfone nanofibers loaded with human cord blood-derived CD34 + cells (hereinafter referred to as CD34 + cells) homing in the wound site after systemic administration and significantly accelerating wound closure. Wound bed NF- κ B and its downstream effector molecule TNF- α, IL-1β And the sustained pro-inflammatory activity of IL-6 decreased. In addition, improved granulation tissue formation increased collagen deposition and myofibroblasts, and decreased MMP-1 expression was observed (Kanji et al., 2019).

Gellan ha sponge hydrogels loaded with hASCs were pre-cultured in selected standard neurogenic conditioned media and found to be able to generate structures that control angiogenesis and inflammation and stimulate new innervation (da Silva et al., 2017). Wound tissue wrapped in ADSC-loaded silk fibroin chitosan film is almost redeveloped at a position close to normal tissue, and this process seems to depend on the similarity between ADSC and skin stem cells (Wu et al., 2018).

Kaisang et al. (2017) reported that injectable Pluronic F-127 hydrogel encapsulated with adipose stem cells (ADSC) as a drug delivery system to enhance the healing of diabetic wounds. Compared with untreated wounds, vascular endothelial growth factor, messenger RNA expression level of key angiogenic growth factor, transforming growth factor in ADSCs-pluronic-f127 hydrogel treated wounds- β1 and key wound healing GF levels were enhanced.

Fibrinogen and collagen I, respectively, was incorporated into the shell and core of nanofibers to mimic the sequential appearance of fibrinogen and collagen I during wound healing. This biomimetic coaxial scaffold significantly promotes the immunoregulatory paracrine of ASC. Incubation of macrophages with ASC conditioned medium confirmed the enhanced immune regulation of ASC on biomimetic coaxial scaffolds by enhanced M1 to M2 polarization of macrophages (Sun et al., 2021).

###### 3.3.2.2.3 Promote the delivery of stem cells in a 3D environment

ADSCs cultured in a 3D environment has a higher level of proangiogenic ability (Im et al., 2021). Therefore, some studies have designed 3D nanofiber scaffolds, whose size, depth, and shape can be adjusted to adapt to different wound conditions. BMSCs were loaded on the nanofiber scaffolds. The 3D scaffolds loaded with BMSCs could enhance the formation of granulation tissue, promote angiogenesis and promote the deposition of collagen. In addition, this scaffold inhibits the formation of M1-type macrophages and the pro-inflammatory cytokines IL-6 and TNF- α. And promotes the formation of M2-type macrophages and the expression of anti-inflammatory cytokines IL-4 (Chen S. et al., 2020).

Using the electrochemical deposition method to load hADSCs onto collagen, the composite biomaterials formed have high tensile strength, high porosity, excellent biocompatibility, and cell proliferation ability (Edwards et al., 2018). In addition, a human epidermal growth factor curcumin bandage bio couple (EGF curb) loaded with MSCs (MSCs EGF curb) was used at the injured site for wound healing in diabetes. Culturing MSCs on EGF curb enhanced the viability of MSCs and their association with pluripotency and self-renewal (OCT ¾, Sox2 and Nanog). *In vivo* experiments have found that this new type of MSC-loaded dressing can significantly enhance wound closure by increasing granulation tissue formation, collagen deposition, and angiogenesis (Mohanty and Pradhan, 2020).

As a kind of biocompatible dressing, PDMS has a certain clinical value. It has a certain strength and can maintain 3D structure during cell spreading. However, its hydrophobicity and permeability are poor, so it is not feasible to be used for wound repair. However, some studies have made PDMS into a material with a 3D concave surface by photolithography. When this material is used alone, wound healing has not proved. However, after ADSC is added, its 3D structure can make ADSC in a spherical state for a long time (7d), which enhances its ability of proliferation and migration, increases the expression of therapeutic growth factors, and thus significantly improve wound healing (Jeong et al., 2022).

###### 3.3.2.2.4 Promote the action of active components in cells

Cell components are the root of wound regeneration. After wound formation, different epidermal stem cells recruited from different skin regions need to cooperate with other cell types (including fibroblasts and immune cells) to ensure effective and synergistic wound healing. This process depends on the activation, migration, and plasticity of these cell components during tissue repair (Dekoninck and Blanpain, 2019). Only using cells to act on the wound has a certain effect, but the activity of stem cells is low, and the ability of proliferation, differentiation, and migration is inhibited. And biomaterials can provide an improved microenvironment and three-dimensional growth scaffolds for them, and try their best to simulate the physiological state.

The soluble growth factors and cytokines released by MSCs are combined with the curcumin complex bandage designed previously (Fromer et al., 2018), and EGF that constitutes the bandage can also help maintain MSC proliferation, stemness, and self-renewal. The synergistic effect of MSC and curcumin allows the complex bandage that can promote wound healing to be upgraded again (Mohanty and Pradhan, 2020). The long-term effect needs to be achieved by the slow release of the active ingredient, which combines the collagen binding domain (CBD) with SDF-1 α And VEGF were specifically fused, and the sustained release of the two recombinant proteins from the collagen scaffolds was successfully observed. Meanwhile, when cbd-vegf and cbd-sdf-1 α When CO modified scaffolds were implanted into the skin wound model of diabetes rats, they not only showed synergistic effects in promoting angiogenesis, but also reduced inflammation in the short term (Hu Y. et al., 2021).

##### 3.3.2.3 Loading active factor

At first, researchers used the modified double lotion method to prepare recombinant human epidermal growth factor (rhEGF) nanoparticles with poly (lactic acid co glycolic acid) as the carrier. The diameter of rhEGF nanoparticles is about 193.5 nm (diameter), and the particle size distribution is uniform and dispersible. The encapsulation efficiency was 85.6% and the release of rhEGF lasted for 24 h. The composite can promote fibroblast proliferation, and its controlled release enhances the effect of rhEGF to stimulate cell proliferation and shorten wound healing time (Chu et al., 2010).

Effects of sponge based wound dressings made of HA and collagen and encapsulated *in vivo* with epidermal growth factor (EGF). EGF loaded sponge wound dressings promoted epithelialization to a greater exten (Kondo et al., 2012). By gathering (ε-The amine terminated block copolymer composed of caprolactone [PCL] and poly (ethylene glycol) [peg] and PCL was electrospun into biocompatible nanofibers with functional amine groups on the surface through peg linker. Surface chemical binding of EGF to nanofibers. When human primary keratinocytes were cultured on EGF conjugated nanofibers, the expression of specific genes was significantly increased. Immunohistochemical staining results showed that EGF receptor (EGFR) was highly expressed in the EGF nanofiber group (Choi et al., 2008). Recombinant human epidermal growth factor (rhEGF) - bound polyurethane foam (PUF) is time and concentration dependent and accelerates wound healing by promoting wound contraction, epithelial regeneration, collagen deposition and the formation of skin appendages (Pyun et al., 2015). Recently, the nanostructured lipid carrier gel formulation of recombinant human thrombomodulin improved wound healing in diabetes by local administration (Hsueh et al., 2021).

Poly (ether) carbamate polydimethylsiloxane/fibrin scaffolds loaded with VEGF and bFGF (scaffolds/GF loaded NPs) poly (lactic acid co glycolic acid) (PLGA) nanoparticles, the application of scaffolds on full-thickness dorsal skin wounds significantly accelerated wound closure on day 15 (Losi et al., 2013). Another material of rhEGF loaded poly (lactic acid co glycolic acid) (PLGA)–alginate microspheres (MS) showed a statistically significant reduction in wound area on days 7 and 11 in the *in vivo* experiment, which was completely re epithelialized by day 11 and the inflammatory process subsided earlier (Gainza et al., 2013). Chitosan and hyaluronic acid are made into composite sponges, which are loaded with fibrin nanoparticles containing VEGF. After evaluating the characteristics of the material itself such as porosity, expansion rate, biodegradability, mechanical properties and hemostatic ability, researchers found that VEGF can be released in high concentration within 3 days after the attachment of the material and induce angiogenesis (Mohandas et al., 2015). Similar studies also included PLGA nanofibers encapsulated with PDGF, vancomycin and gentamicin 115. While the administration of VEGF encapsulated in PLGA nanoparticles (PLGA-VEGF NPs) will promote rapid healing due to the sustained and combined effects of VEGF and lactate (Chereddy et al., 2015).

The prepared PVA solution was mixed with CTGF solution to obtain a 6% w/v PVA solution containing 0.1% w/w CTGF. For PLA shells, a 10% PLLA solution was prepared in a DCM/DMF (1:9 ratio) mixed solvent system. The electrospun pva-pla hybrid membrane coated with connective tissue growth factor prepared by electrospinning showed high cell proliferation and migration ability of fibroblasts, keratinocytes and epithelial cells with potential angiogenesis (Augustine et al., 2019).

In addition, nanofiber biodegradable drug loaded films with sustained release of recombinant human platelet-derived growth factor (rhPDGF BB) to repair diabetes wounds have been developed. RhPDGF BB and polylactic acid co glycolic acid (PLGA) were mixed in hexafluoroisopropanol, and then the solution was electrospinned into biodegradable membranes to equip nanofiber membranes. Based on the formation of Schiff base bond, ODEX/HA-AMP/PRP hydrogel was prepared by mixing oxidized dextran (ODEX), ha-amp modified by antimicrobial peptide and PRP under physiological conditions, which has obvious bacteriostatic circle. It has significant antibacterial activity against *Staphylococcus aureus* and *Pseudomonas aeruginosa*, and inhibits proinflammatory factors (TNF-α, IL-1β and IL-6), enhancing anti-inflammatory factor (TGF- β1) And the production of vascular endothelial growth factor (VEGF) (Wei et al., 2021).

Polylactic acid glycolic acid/collagen nano-scale dressing, and surface functionalization with wound healing peptide, loaded chitosan nano and micro scale particles to form extracellular matrix (ECM)-like structure with bionic function. The developed dressings have good cell compatibility with keratinocytes and fibroblasts and enhance their cell proliferation and migration ability *in vitro*. Experiments in streptozotocin induced diabetes mouse model showed that bioactive peptides released by chitosan particles could shorten the inflammatory phase and promote neovascularization. The supportive nanoscale matrix promotes increased collagen deposition in the wound bed, thereby accelerating the complete healing process through massive tissue regeneration and functional recovery. The results showed that the nanoscale mat rich in nano/particles showed the potential as an effective wound repair dressing for diabetes wounds (Nanditha and Kumar, 2022).

Horseradish peroxidase (HRP) catalyzed spray gelatin gel (GH) loaded two types of chemokines [i.e., macrophage inflammatory protein 3A (MIP-3a) and interleukin-8 (IL-8)] by *in situ* crosslinking. IL-8, MIP-3 without affecting GH swelling rate and mechanical stiffness α Release from GH and maintain biological activity, and finally significantly promote wound healing in diabetes by stimulating collagen deposition and neovascularization/re-epithelialization (Wei et al., 2021).

The antioxidant thermoresponsive hydrogel is composed of poly (poly (ethylene citrate co-n-isopropyl acrylamide) (PPCN) through sequential polycondensation and free radical polymerization. SDF-1 is blocked by gel of ppcn + SDF-1 solution above its lower critical solution temperature (LCST), and its release and biological activity are measured. Finally, under the action of accelerated granulation tissue formation, epithelial maturation, and the highest density of perfusion vessels, The repair of 6 mm diameter wounds in mice was completed in 24 days (Zhu et al., 2016).

Heparin-bound epidermal growth factor (HB-EGF) has a positive effect on wound contraction, epithelial regeneration and collagen deposition due to its affinity for heparin-bound growth factor. In the highly sulfated heparin-like polysaccharide 2-N, 6O sulfated chitosan (26SCS)- doped poly (lactic acid co glycolic acid) scaffolds (S-PLGA), 26scs has strong scavenging activity against superoxide radicals. *In vivo* experiments, the sustained release of HB-EGF induced by 26scs and the migration of glial forming cells after ROS removal can also be observed (Zhang X. et al., 2018).

Chitosan, silk fibroin, and PRP (CBPGCTS-SF@PRP) The composing self-healing and injectable hydrogels can protect PrP from enzymatic hydrolysis, sustainably release PrP, and enhance the chemotaxis of mesenchymal stem cells (Qian et al., 2020).

A natural polysaccharide-based hydrogel matrix was prepared using green algae sulfide polysaccharides, chitosan, dopamine (DPA), and silver nanoparticles (Ag NPs). Human umbilical cord mesenchymal stem cells lyophilized powder (HUC MSCs) was loaded into hydrogels to develop a new type of wound healing material for chronic diabetes (UC-DPA-Ag@hUC-MSCs). The resulting hydrogel has sufficient mechanical properties, swelling ability, adhesion, antioxidant, antibacterial ability, and the ability to promote cell proliferation and migration. The *in vivo* wound healing of the wound model of type II diabetes mice showed that HUC MSCs loaded with UC DPA Ag hydrogel could effectively accelerate wound healing. This advanced hydrogel provides a simple and effective method for chronic wound management in diabetes (Ren Y. et al., 2022).

Injectable BG/sodium alginate (BG/SA) hydrogel loaded with MMP9 SINP can significantly accelerate the healing process of full-thickness resected wounds in diabetes rats by reducing MMP-9 expression, improving collagen synthesis and enhancing wound angiogenesis. Injectable bioglass/sodium alginate (B/SA) hydrogel loaded with MMP9 SINP significantly accelerated the healing process of full-thickness excised wounds in diabetes rats by reducing the expression of MMP-9, improving the accumulation of collagen and enhancing angiogenesis in the wounds (Li et al., 2022).

##### 3.3.2.4 Loading ions

###### 3.3.2.4.1 Ag^+^

Silver nanoparticles have strong wound-healing potential due to their well-known antibacterial activity. The mixed solution of polyethylene glycol (PEG) and chitosan reduces silver nitrate to convert silver ions into silver nanoparticles (Ag NPs), to obtain chitosan peg prepolymer containing Ag NPs. After that, the prepolymer solution is crosslinked by glutaraldehyde to form a hydrogel. The hydrogel impregnated with Ag NPs has higher porosity, higher swelling degree and, higher water vapor conversion rate (WVTR). In addition, Ag NPs have the antibacterial and antioxidant abilities, so the obtained polymer is beneficial to the wound healing of diabetes (Masood et al., 2019). Chitosan dextran hydrogel loaded with Ag nanoparticles also has a broad-spectrum antibacterial effect, promoting granulation tissue formation, fibroblast migration and angiogenesis (Shi et al., 2019).

A wound dressing composed of chitosan, crosslinked gelatin/polyvinylpyrrolidone, and embedded silver nanoparticles were prepared by solution casting method. The films were characterized by FTIR, SEM and TGA. Glutaraldehyde (0.5%) was used for cross-linking of membrane modules, and was associated with a 7-fold improvement in mechanical properties, 28% hydrolysis stability, 3-fold reduction in thickness and morphological roughness. This developed membrane can serve as a promising and cost-effective system to combat severe diabetes and burn wound infections (El-Aassar et al., 2021).

In addition, the combination of nanosilver, chitosan, and hyaluronic acid has also been reported, and its antibacterial mechanism has been studied in depth: ①nAg damages the bacterial cell wall and interferes with the proteins involved in the formation of membrane potential; ②The proton gradient necessary for electron transfer to establish oxidative phosphorylation is disrupted; ③Membrane damage and permeability change. Due to the in-depth discussion of the above mechanisms, the clinical status of anionic antibacterial dressing was established (Anisha et al., 2013).

The preparation of biocompatible and multifunctional self-assembled hydrogels using polymer nanoparticle interactions is a simple and universal approach for effective chronic wound treatment. Mixed silver lignin nanoparticles play two roles: ① structural role, acting as a cross-linking node in the hydrogel and giving it shear thinning (the ability to flow under the applied shear stress) and self-healing characteristics; ② Function: it has strong antibacterial and antioxidant activities. Thiolated hyaluronic acid and Ag@LigNPs The nanocomposite hydrogel produced by *in situ* self-assembly of can simultaneously inhibit the main factors of wound chronicity, i.e., overexpressed harmful proteolytic enzymes and oxidase, and high bacterial loaded (Pérez-Rafael et al., 2021).

###### 3.3.2.4.2 Cu^2+^

Cu ion is a basic element with a long history of use in humans (Gopal et al., 2014). It is involved in many processes related to wound healing, including the induction of vascular endothelial growth factor, angiogenesis, and the expression and stabilization of extracellular skin proteins such as keratin and collagen (Marelli et al., 2015). Cu ion is also a well-known antibacterial agent, which can promote healing by reducing the possibility of wound infection (Amna et al., 2014). The increase in nonphysiological concentrations of copper ions may be toxic because these ions interfere with the homeostasis of other metals, disrupt DNA, and generate reactive oxygen species that may adversely affect proteins, lipids, and nucleic acids. However, if copper ions are slowly released from the warehouse placed at the desired location, the toxicity may be mitigated (Guo et al., 2013). Metal-organic frameworks (MOFs), also known as porous coordination polymers, are crystalline porous materials composed of inorganic metal ions or clusters connected by polydentate organic ligands (Lu et al., 2014). The citric acid saline gel can be safely absorbed in the thermal response of oxidation resistance. On the basis of the above research, Xiao et al. (2017) Developed a composite scaffold combining copper ions with a basic framework and made a hydrogel.

Firstly, poly (ethylene citrate) acrylate prepolymer (PPcac) was prepared by polycondensation of citric acid, PEG and glycerol 1,3-diglycidyl diacrylate. Subsequently, PPcac was reacted with pre purified NIPAM by radical polymerization overnight, using AIBN as a radical initiator. The reaction product PPCN was obtained by precipitation and purification with ether. Copper acetate monohydrate (0.15 g, 0.75 mmol) dissolved in distilled water (2 ml) was added dropwise to h3btc (0.11 g, 0.5 mmol) dissolved in ethanol (2 ml), and then stirred at room temperature for 20 min to form a gel-like dark Turquoise suspension. The suspension was then centrifuged and the precipitate was washed twice with ethanol/water (1:1, V/V) solution to obtain purified HKUST-1. Hkust-1nps were added to PPCN solution (100 mg/ml) and the copper concentration was 0.1 M under vortex at room temperature. The solution was directly placed on the QFDE sample tray, heated to solid at 45°C, and then rapidly frozen. Further functional validation found that h-hkust-1 hydrogel can protect nanoparticles from decomposition and slowly release copper ions, thereby reducing apoptosis and cytotoxicity, enhancing dermal cell migration, and improving wound closure rate (Xiao et al., 2017).

###### 3.3.2.4.3 Ce^2+^

Cerium is a rare Earth element, which is widely used in industrial fields such as catalysts, fuel additives, and colored components incorporated into glass. Ce^4+^ and CeO2 show high antibacterial activity. Silver, copper, zinc, and gallium have been incorporated into a bioactive glass (BG) to give it antibacterial ability. Direct contact with BG may adhere to the wound and cause adverse reactions such as wound tearing. BG-loaded hydrogels can avoid adverse reactions caused by BG alone (Cheng et al., 2021). Photo cross linkable and biodegradable gelatin methacrylate (gel MA) as the framework of hydrogel, loaded with cerium containing bioactive glass nanoparticles, can significantly reduce the colony number of *Staphylococcus aureus* and *Escherichia coli*. *In vivo* experiments verified its role in promoting wound healing (Chen et al., 2021).

Diabetes wounds have extremely complex microenvironments of hyperglycemia, hypoxia, and high reactive oxygen species (ROS). Based on the co-assembly of CE-driven special double ligands (alendronate and 2-methylimidazole) and glucose oxidase (GOx), a glucose/ROS cascade responsive nanoenzyme was developed (CHA@GOx) Yes, for wound treatment of diabetes. It has superoxide dismutase and catalase mimetic activities and can effectively remove excess ROS. In particular, it can catalyze the excessive hydrogen peroxide produced by glucose oxidation reaction to generate oxygen, regulate the oxygen balance of the wound, reduce the toxic and side effects of GOx, and achieve the purpose of synergetic repair of diabetes wounds. *In vitro* experiments showed that, CHA@GOx It contributes to the migration of mouse fibroblasts and promotes the formation of human umbilical vein endothelial cell tubes. *In vivo* experiments, it can induce angiogenesis, collagen deposition, and epithelial reformation (Yu et al., 2022).

###### 3.3.2.4.4 Zn^2+^

Microporous and flexible chitosan-based hydrogel composite bandages loaded with ZnO nanoparticles showed improved blood coagulation, controllable degradation, swellingand good antibacterial activity. The 80% porosity can absorb a large amount of exudate and improve cell viability, thus promoting wound healing while inhibiting bacteria (Kumar et al., 2012).

After methacrylation of simulated natural glycosaminoglycans κ- When carrageenan (Kama) is used as the hydrogel matrix, polydopamine of different concentrations is loaded to improve the mechanical, antibacterial and cellular properties. L-glutamic acid promotes wound healing. The composite material has good elasticity and adhesion as a whole, and can effectively coagulate blood. It also ensures that the viability of incubated cells is still 95% after 3 days. *In vivo* experiments found that it accelerated wound healing by promoting granulation tissue proliferation (Tavakoli et al., 2020).

Epigallocatechingallate (EGCG) modified zinc oxide quantum dots hydrogel (ZnO-EGCG@H), it is used to treat the delayed wound of diabetes. EGCG was successfully combined with ZnO quantum dots through phenol hydroxyl groups and formed a coordination bond with Zn atoms on ZnO quantum dots. Then, the obtained ZnO EGCG was packaged in hydrogels as a dual-purpose nano reagent to promote angiogenesis and epithelial regeneration by enhancing the expression of VEGF and EGF. In addition, hydrogel, as an excellent drug delivery system, provides continuous water and high local concentration of ZnO EGCG, enabling ZnO-EGCG@H By reducing inflammatory factors (TNF-α, IL-6) and the production of various antibacterial mechanisms including ROS to accelerate the wound healing of diabetes and provide ideal biomaterials for the wound treatment of diabetes. ZnO-EGCG@H After 15 days of treatment, the lesion closure rate of the rats was 96.3%, which was significantly better than that of the control group (65.4%). The safety experiment shows that, ZnO-EGCG@H It has reassuring biocompatibility and is harmless to important organs (Yin et al., 2022).

Researchers have developed a new healing hydrogel based on histidine, a natural dietary essential amino acid that is important for tissue formation. Amino acids are crosslinked with zinc ions (Zn^2+^) and sodium alginate (SA) through dynamic coordination bonds and hydrogen bonds respectively to form histidine-SA-Zn^2+^ (HSZH) hydrogels with good injectability, adhesion and biocompatibility and antibacterial properties. The application of this double dynamic bond crosslinked hydrogel accelerated the migration and angiogenesis of skin related cells *in vitro*. *In vivo* experiments, the wound was completely repaired within about 13 days, while the healing process of the control group took about 27 days. This weakly crosslinked material based on tissue-friendly small molecules can cure wounds more effectively than the highly crosslinked material based on long-chain polymers (Yao et al., 2022b).

##### 3.3.2.5 Loading gas

###### 3.3.2.5.1 NO

Since 1987, the role of no *in vivo* has been gradually clarified, especially its ability to control cell proliferation and apoptosis, promote angiogenesis induced by growth factors and promote wound healing (Miller and Megson, 2007). However, this feature depends on the site and concentration of no generation. Current research shows that the surface flux of no is 0.5–4.1 × 10^−10^ mol cm^−2^ min^−1^. However, due to the extreme reactivity, short half-life and short diffusion distance of no, artificial application is very difficult (He et al., 2019). A particle based no release material has been developed, which can produce physiologically relevant levels of no for a long time without any toxic and side effects (Lautner et al., 2016). The NO donors-nitroso-n-acetyl-d-penicillamine (SNAP) was encapsulated in 50:50 (PLGA) and microspheres were prepared using oil in water solid lotion solvent evaporation method. Snap was slowly released for more than 10 days, while release continued for more than 4 weeks by using ester terminated PLGA (Mw = 38,000–54000). The presence of copper ions and/or ascorbate in the solution is effective for decomposing the released no donor and obtaining sustained no release. It was also demonstrated that light could be used to induce the microspheres to release no rapidly within several hours. These new microsphere formulations can be used for site-specific administration and treatment of diseases related to dysfunction of endogenous NO production. The microparticles reported here can eventually be injected into many locations in the body or incorporated into creams or hydrogels to generate wound healing patches that can be used to treat various types of wounds, including ulcers related to diabetes (Lautner et al., 2016).

Gelatin methacrylate (gelma) has been proven to be a highly cell friendly, cell adhesive, and inexpensive biopolymer for various tissue engineering and wound healing applications. In this study, the nitric oxide (no) donor SNAP was incorporated into the highly porous gelMA hydrogel patch to improve cell proliferation, promote rapid cell migration and promote wound healing in diabetes (Zahid et al., 2021).

A new type of porous metal organic framework (MOF) microneedle (MN) patch can realize the delivery of photothermally responsive nitric oxide (no) to promote the wound healing of diabetes. Since the copper-benzene-1,3,5-tricarboxylic acid copper (HKUST-1) MOF that can carry no is encapsulated by graphene oxide (go), the resulting NO@HKUST-1 @Go microparticles (NHGS) can promote the controlled release of no molecules in the near infrared ray (NIR) photothermal response. When these NHGS are embedded in porous pegda-mns, the porous structure, larger specific surface area and sufficient mechanical strength of the integrated MNS can promote more accurate and deeper delivery of no molecules to the wound site (Yao et al., 2022a).

###### 3.3.2.5.2 O_2_

Hyperbaric oxygen therapy is a commonly used chronic wound treatment method in clinic at present. The oxygen concentration in the body is increased through the whole-body treatment of hyperbaric oxygen chamber. There are studies on hyperbaric oxygen therapy for systemic application, combined with WJ-MSCs based on IM (a commercial wound care device, integramatrix wound dressing, which provides the ECM for cell invasion and capillary growth), to promote wound healing in diabetes mice *in vivo* experiments (Peña-Villalobos et al., 2018). However, due to the low vascular density around the chronic wound and slow local blood circulation, the treatment effect is not satisfactory, and there may be concurrent problems such as oxygen poisoning. Therefore, scientists think of local oxygen therapy by local administration. Some studies have used frozen gel technology to make it by adding calcium peroxide (CPO) to the antioxidant polyurethane (PUAO) scaffolds. PUAO-CPO frozen gel attenuated ROS and showed sustained oxygen release over a period of 10 days. *In vitro* analysis showed that they could maintain H9c2 cardiomyocytes under hypoxia, and the cell viability was significantly better than that of common polyurethane (PU) scaffolds. In addition, *in vivo* studies using an ischemic flap model showed that oxygen releasing frozen gel scaffolds were able to prevent tissue necrosis for up to 9 days. This approach can be used to develop oxygen releasing biomaterials with sustained oxygen delivery and reduced production of residual ROS and free radicals due to ischemia or oxygen production (Shiekh et al., 2018). After that, there were studies using 7%PCL +2% sodium percarbonate (SPC) at 19 kv voltage and 50 μ L flow rate, and found that O_2_ in the electrospun sheet can be sustained for up to 10 days, and the cells seeded on the SPC can express higher levels of HIF-1 at the gene and protein levels α. *In vivo* experiments, it shows a strong angiogenic effect and forms a relatively dense ECM (Zehra et al., 2020).

However, it seems that the oxygen release in 9–10 days is still insufficient, so scientists based on the oxygen release microspheres (ORM) and the oxygen generation system of injectable, rapid gel and reactive oxygen species (ROS) scavenging hydrogel (Ross gel) (Guan et al., 2021). The microspheres rapidly released enough oxygen to support the cells to survive under hypoxia and maintain oxygen release for at least 2 weeks. Unlike most current oxygen production systems that first release toxic H_2_O_2_ into the tissue environment and then release oxygen through its decomposition, the designed ORM directly releases oxygen, clears ROS, and avoids its damaging effect on cells. The continuous oxygenation of released oxygen to skin cells promotes the survival, migration and paracrine of skin cells and the formation of endothelial lumen under hypoxia *in vitro*. In addition, after effectively increasing intracellular oxygen content and adenosine triphosphate content, extracellular signal regulated kinase 1 and heme oxygenase 1 signaling (ERK 1/2 and HO-1) were activated. Meanwhile, *in vivo* experiments, it was found that sustained oxygenation and ROS clearance in diabetes wounds stimulated the expression of angiogenic growth factors and angiogenesis (Guan et al., 2021).

## 4 Biomaterials development towards intelligence

We expect that the accumulation of single active ingredients can achieve synergistic effect, but most attempts are contrary to our expectations. More attempts have been made to design more intelligent composite materials.

### 4.1 PH sensitivity

PH responsive hydrogels were prepared by Schiff base crosslinking using phenyl boron modified chitosan (CSPBA), polyvinyl alcohol (PVVA) and benzaldehyde terminated peg, and insulin and fibroblasts were encapsulated into the hydrogels at pH = 7.4. When the pH decreases, the Schiff base is unstable, and the phenyl boron group preferentially binds to glucose. In addition, in high-level glucose water, the modulus of the hydrogel decreases, which means that insulin molecules are released through the swollen hydrogel matrix. Due to the acidic environment and high glucose level in the wound area of diabetes, this intelligent responsiveness is what we want to see (Zhao et al., 2017).

Traumatic multidrug resistant (MDR) bacterial infection is a fatal threat to the public. To combat MDR bacteria, a bifunctional pH sensitive hydrogel based on peptide DP7 (VQWRIRVAVIRK) and oxidized dextran (DP7-ODEX hydrogel) was developed. As an antimicrobial peptide, DP7 can synergize with many antibiotics. When ceftazidime was added to DP7-ODEX hydrogel, it showed obvious advantages in MDR inhibition of *Pseudomonas aeruginosa*. This hydrogel made of the bifunctional peptide DP7 can kill multidrug-resistant bacteria colonizing the wound bed and promote scar free wound healing (Wu et al., 2022).

Under alkaline conditions, graphene is reduced to graphene oxide (pGO) by PDA, and pGO is dispersed in chitosan CS/silk fibroin SF. After double crosslinking, pGO gel is obtained. The pGO in the gel can clear excessive ROS and promote wound regeneration through physiological and electrical signal transmission that promotes cell growth (Tang et al., 2019).

Gini flat cross linked chitosan (CHT)/gelatin (GEL) scaffolds were fabricated by lyophilization and loaded with platelet rich plasma (PRP). Human dermal fibroblasts were seeded on the scaffolds, and then polychromatic light in the NIR was applied to the scaffolds to activate platelets and stimulate fibroblasts (photoactivation, PAC). Thus, fibroblasts were chemically and physically stimulated by PRP and light, respectively. It was found that the expression of laminin and collagen 4 was up-regulated, and the angiogenesis related PDGF and VEGF were also significantly increased under the action of PRP and light (Koyuncu et al., 2022). However, this complex system also means that biomaterials have also changed from single to complex, from the combination of simple biomaterials and some ingredients that promote wound healing to a bionic system that strives to restore the normal wound healing mode (Bulutoglu et al., 2022). Nano ZnO was loaded into the hydrogel to kill microorganisms. Paeoniflorin encapsulated micelles with ROS responsive properties were immobilized on the framework of hydrogels by Schiff base bonds for low pH and ROS stimulated angiogenic activity. The continuous responsiveness of the novel hydrogel can intelligently rescue the harmful microenvironment in refractory wounds. This highly biocompatible hydrogel significantly promotes the healing of chronic infected diabetes wounds *in vitro* and through continuous hemostatic, microbial killing and angiogenic activities. This microenvironment responsive hydrogel loaded with nzno and PF encapsulated micelles has great potential as a site-specific dual response delivery platform for the treatment of refractory, chronic infected diabetes wounds (Guo et al., 2022). Cellulose nanofibers (CNFs) obtain pH responsiveness through acro amino hyperbranched polyamines (HBP-NH_2_) and simultaneously load indocyanine green (ICG) to achieve temperature responsiveness. In addition, the 3D cage structure of the material itself provides high loading capacity of doxorubicin and ICG, thus achieving multi responsive composite materials (Zhao et al., 2021). The introduction of exogenous factors such as visible light and heat has broadened the design idea of wound dressing (Yanina et al., 2017).

### 4.2 Optical responsiveness

New glycerol monoester based thermosensitive matrix as wound management system. First, an appropriate portion of glycerol monooleate (GMO) and glycerol monostearate (GMS) were mixed to provide a thermoresponsive matrix (gmo-gms, GG). Subsequently, in order to improve the photothermal response and antibacterial properties, silver nanoparticles (Ag)–modified reduced graphene oxide (RGO) nanocomposites (RGO Ag) were added to the GG matrix to obtain (GG RGO Ag). According to the systematic study of uninfected, infected and diabetes wound models, the phase transition of GG RGO Ag can be triggered by applying NIR laser to release Ag for sterilization as required. More importantly, this smart GG substrate can also promote the production of vascular endothelial growth factor protein, thus serving as a multi effect wound management system defined by NIR(Jin et al., 2021).

Graphene quantum dots modified luminescent porous silicon material has become a new dressing. The luminescent porous silicon carrying EGF and insulin was embedded in the chitosan film. Under the effect of fluorescence resonance energy transfer, the dressing was red, H2O2 triggered Si oxidation, and at the same time, the drug was released, while the PL of gqds was restored, and the dressing was blue. *In vitro* and *in vivo* results showed that smart dressing enhanced cell proliferation and migration and significantly cured diabetes wounds (Cui et al., 2021).

And exogenous speed limiting intervention measures can better realize the intellectualization of dressing. PLGA is used as a scaffold to encapsulate Mxene nanosheets and mesoporous silica nanoparticles containing VEGF. After that, dopamine hyaluronic acid hydrogel is used as a shell to realize photothermal conversion through NIR, so as to release an appropriate amount of VEGF from the Mxene nanofiber skeleton to promote angiogenesis. DA generates H_2_S to induce macrophage polarization. The thermal effect of NIR itself promotes local blood circulation, and multiple effects combine to achieve controllable release of effective substances, thereby promoting wound healing (Jin et al., 2022).

Biocompatible Cu_3_SnS_4_ NFs nanosheets are prepared by a simple and low-cost manufacturing process. These NFs can be activated by visible light, leading to visible light mediated photocatalytic production of a large amount of reactive oxygen species (ROS). In addition, plasma Cu_3_SNS_4_ NFs exhibited strong NIR absorption and high photothermal conversion efficiency of 55.7%. The novel combination promotes endothelial cell angiogenesis and collagen deposition, thus accelerating wound healing. In addition, their inherent local surface plasmon resonance effect makes them active substrates for surface enhanced Raman scattering (SERS) imaging and SERS labeled bacterial detection (Yang et al., 2022).

### 4.3 Internal environment responsiveness

The stable and uniform distribution of polydopamine reduced graphene oxide enables the scaffolds to have stable electrical and mechanical properties even after long-term immersion. Due to its unique biomimetic structure and tissue affinity, the scaffold further acts as an “electronic skin,” transmitting endogenous bioelectricity by absorbing wound exudates, and promoting the treatment of diabetes wounds (Wang J. et al., 2022). Injectable sodium alginate/Bioglass (SA/BG) composite hydrogel is used to carry SA microparticles containing cell conditioned medium (CM) (SA-CM), including pirfenidone (PFD) polylactic acid (PLGA) microspheres. This multilayer injectable gel system delivers bioactive molecules in sequence. 1-3d regulates host inflammatory response, 2-7d promotes vascularization and granulation tissue formation, 8-20d releases PFD, and prevents regenerative skin fibrosis and scar formation (Ma et al., 2020).

Another custom dressing was derived from antibacterial nanoparticles containing recombinant human type III collagen (rh Col III) (PDA@AgNPs), first released PDA@AgNPs Rapidly kill *Staphylococcus aureus* and *Escherichia coli*, and subsequently, customized rh col III was used to promote the proliferation and migration of fibroblasts and endothelial cells (Hu C. et al., 2021). The composite material based on rh col III and naproxen (nAP) is composed of polylactic acid (PLGA) nanoparticles combined with hyaluronic acid (HA) microneedles (MN). It can also deliver effective drugs in order according to the wound conditions. It is worth mentioning that rh col III is synthesized based on Gly483-Pro512 fragment and has strong cell adhesion (Long et al., 2022).

### 4.4 Temperature responsiveness

A recombinant fusion protein, which contains a rage (vRAGE) binding domain linked to elastin like polypeptide (ELP), can self assemble into a coacervate at about 30–31°C. The size of the condensed layer is related to the concentration and temperature, and the range is 500–1600 nm. VRAGE-ELP reversed several age-mediated changes in cultured human umbilical vein endothelial cells, including a decrease in the number of viable cells, an increase in the level of reactive oxygen species (ROS), and an increase in the expression of the pro-inflammatory marker intercellular adhesion molecule-1 (ICAM-1). vRAGE-ELP can stably exist for 7 days *in vitro*. This coagulation system that locally delivers competitive ages inhibitors has the potential to treat diabetes wounds (Kang et al., 2021).

A temperature resistant (−20–60°C) antibacterial hydrogel dressing consists of polyacrylamide, gelatin and ε- Poly lysine is assembled. Due to the binary solvent system of water/glycerol (Gly), the resulting hydrogel (G-PAGL) showed good heat resistance and freezing resistance, and showed long-lasting and extensive antibacterial activity against Gram-positive and Gram-negative bacteria. It is satisfactory that the double network (DN) G-PAGL hydrogel dressing can effectively promote the healing of DFUs by accelerating collagen deposition, promoting angiogenesis and inhibiting bacterial growth (Liu et al., 2021).

## 5 Application and analysis of composite biomaterials used in clinical trials

Refer to https://clincialtrials.gov by taking wounds and different types of materials (hydrogels, scaffolds, films, nanofibers) as the search keywords. It is not difficult to see that the number of relevant clinical studies is small compared with basic research, and the quality of published articles is also mixed. The purpose of basic research is ultimately to apply the research results to the clinic. Therefore, finding a suitable clinical transformation path is also a problem that researchers must consider.

### 5.1 Status of clinical trials

Most of the clinical research on biomaterials is concentrated in the past 5 years, which depends on the progress of basic research. However, the conclusions obtained in the basic research may not be directly applicable to the human body.

#### 5.1.1 Fundamental deviation of basic research

The biocompatibility and effectiveness of some classic degradable biomaterials have been confirmed in clinical trials, but the next development has experienced a cliff like stagnation. One of the important reasons is that the application of biomaterials has transitioned from a single material to a new stage: composite biomaterials. As we have analyzed in the previous article, biomaterials can be combined with stem cells, active factors and other substances, which is recognized in the academic field, but it cannot be smoothly carried out in clinical transformation due to ethics, diversity of ingredient sources and other reasons.

In addition, most of the animal models selected for basic research are rodents, which have the advantages of wide sources, mature model preparation methods and easy feeding. However, the biggest problem is that its wound healing mechanism is different from that of humans. This problem may fundamentally negate the results of basic research. To solve this problem, scientists used rubber rings to prevent skin contraction to brake the wound. After that, large mammals such as pigs were used for preclinical trials. However, there are still some clinical trials that fail to achieve the desired results in human body, which makes the clinical transformation work stagnate. However, preclinical trials in mammals and primates can still reduce the risk of clinical trials to a certain extent.

#### 5.1.2 Feasibility of basic research

There is no doubt that the ultimate goal of basic research is to apply it to clinical practice. Therefore, in the basic research, the design of the experimental scheme should take the clinical application as the ultimate goal. In order to be convenient, quick and easy to operate, comprehensively study and judge the factors affecting ethics, economy and psychology, and design the experimental scheme with strong transformation ability. Otherwise, it will be of little help to clinical work. The basic research team should communicate closely with the clinic and conduct targeted basic research for the purpose of clinical transformation in combination with the actual clinical needs.

Basic research still focuses on confirmatory research, which verifies the effectiveness and safety of materials through the development of materials and observation of wound healing. However, as a basic research, the verification of material safety and effectiveness is far from enough. We have to figure out which behaviors, which signals, or which pathways are affected by the design and development of various materials. Only by fundamentally clarifying the mechanism can this material be brought to the clinic.

#### 5.1.3 Complexity of clinical situation

For basic research, there are mature models and methods to construct wound healing model system and verify the effectiveness of biomaterials *in vivo* and *in vitro*. However, the complexity of clinical wound is not only due to its location, area and wound environment, but also due to other factors. The patient’s economic ability and many personal conditions, including compliance, are different. It needs to combine various factors to promote the final clinical transformation. From the current clinical research and the publication of high-quality articles, it can be seen that simple operation methods and the intervention of finished product manufacturing companies are the keys to the success of clinical trials. As far as the materials themselves are concerned, the most teams use film materials for clinical research, which may be due to strong operability and good patient compliance. The number of cases included in the current clinical study is generally small, mostly ranging from 20 to 50. In addition to the limitations of the clinical trial itself on the situation of the subjects, the conservative psychology of the subjects and the ability to bear unexpected situations are uncontrollable. In one study, 100 people were included as subjects, but the trial had to be terminated due to too many lost visits. Such cases can be found everywhere. In addition, the protocols, results, endpoint values and follow-up time of different clinical trials are different, and the lack of standardization makes it more difficult to compare the collected data and different types of treatments (Chereddy et al., 2016; Nilforoushzadeh et al., 2017).

### 5.2 Solution

The standardization and rational use of biodegradable biomaterials will be an inevitable trend. Therefore, solving the existing problems is the best way to promote the development of this field.

#### 5.2.1 Develop uniform standards

Under the guidance of the industry association, an expert technical committee and a technical alliance shall be established, and a unified technical standard shall be formulated. Relevant basic research shall be evaluated according to the unified standard, so as to ensure the effectiveness and repeatability of experimental data while ensuring innovation, so that the data can be summarized horizontally and vertically, and the accuracy of data can be rapidly improved, so as to accelerate the transformation of results into clinical practice.

#### 5.2.2 Support work

The funds generated in the process of scientific research should not become the burden of scientific research personnel. After the project design and scheme pass the review, different funds should be provided. In particular, the government can provide funds through insurance, risk investment and other channels to fully guarantee the product quality, medical application safety and accelerate the transformation to clinical practice.

#### 5.2.3 Early enterprise involvement

An important part of promoting clinical transformation is related drug and device enterprises that commercialize and popularize the results. In the early stage of R & D, enterprises are introduced to share and dilute risks, and accelerate the progress of R & D and industrial transformation.

#### 5.2.4 Improve the process system

Promote the rapid advancement of legislative and regulatory procedures through various channels, such as academicians’ suggestions and NPC deputies’ proposals. Improve relevant laws and regulations, accelerate the training of review professionals, and then improve the review speed, so as to form scientific management in this field.

We believe that biomaterials are an excellent direction for wound repair, but the choice of specific programs still needs to be discussed. The combination of biological materials with active ingredients should not be blunt, and the imposition of cells or cellular components on the material does not necessarily work perfectly. For example, loading cells with material results in a decrease in cell loading and the number of adherent cells is affected by factors such as surface area and hydrophilicity of the material surface, which is not worth the loss. In the development and utilization of biomaterials, more attention should be paid to the interaction between materials and cells.

## 6 Expansion of research ideas and future research directions

OxOBand is composed of antioxidant polyurethane (PUAO) as a highly porous frozen gel with continuous oxygen release characteristics, supplemented by adipose stem cells (ADSCs) exosomes. Exosomes engulfed by cells enhanced the migration of human keratinocytes and fibroblasts and increased the survival rate of human neuroblastoma cells under hyperglycemic conditions. Compared with untreated diabetes control wounds, oxoband promoted faster wound closure, enhanced collagen deposition, faster epithelial regeneration, increased neovascularization, and reduced oxidative stress within 2 weeks. This dressing promotes the development of mature epithelial structures, and the morphology of hair follicles and epidermis is similar to that of healthy skin (Shiekh et al., 2020). This method will change the passive to active, so that the cells become “hungry” and take what they want from the materials we provide. Although this sounds like a difficult goal to achieve, it is still a better research direction.

However, whether it is the application of exosomes or various cell active ingredients, the most important thing is to improve its utilization rate while clarifying its effectiveness. Most of the cells and drugs exist in the form of fluids, and the rational use of biomaterials should provide an appropriate “refuge” for the effective substances that are difficult to exist stably for a long time, so that they will not be attacked by the immune system and will not be cleaved due to the unfriendliness of the environment, but will be released at a suitable time to play their due role.

In addition, compared with the use of toxic oxidative cross-linking initiators (such as sodium periodate and silver nitrate) traditionally used, the current research is more inclined to the use of effective extracts in natural ingredients. For example, the natural polyphenol compound tannic acid (TA) achieves near instantaneous (<25 s) hydrogen bond mediated citrate gel. Mussel-based biological adhesives combine antioxidant, anti-inflammatory, and antibacterial activities (3A-TCMBAs). *In vivo* evaluation in the infected full-thickness skin wound model and the rat skin incision model showed that 3A-TCMBAs + NIR treatment could promote wound closure and collagen deposition, increase the collagen I/III ratio at the wound site, and inhibit the expression of pro-inflammatory cytokines. In the early stage, the wound promotes angiogenesis, and in the later stage, the remodeling and degradation of ECM are triggered by platelet endothelial cell adhesion factor-1 to promote scar-free healing of the wound (Wu et al., 2023). This study seems to suggest the development direction of biomaterials in the wound in the future.

There is a relatively clear time window and natural process for wound healing. In different periods, different cells, growth factors, and extracellular matrix play different roles. For example, fibroblasts act as the main component of ECM during the formation of granulation tissue. However, if the process of fibroblasts transforming into myofibroblasts is out of control at the later stage of wound healing, the next pathological process may be induced: hypertrophic scar. Therefore, the superposition of simple effective ingredients may not play a perfect role, but more importantly, it can restore the ability of normal wound healing while treating the effects of some pathological factors (such as infection, biofilm formation, and vascular degeneration) on the wound (Zhang X. et al., 2018). Although external intervention is important, restoring the original healing mode may be a supplement to the normal physiological process.

Despite the remarkable achievements, the treatment of diabetes-related chronic wounds is still very challenging due to the complexity and diversity of etiology and pathogenesis. At present, the bottleneck that is still difficult to break through lies in the difference in animal models. There is a great difference in the process of skin healing between mice and humans, but the mouse model is still difficult to replace (Wang L. et al., 2022). Therefore, establishing a mouse animal model with less difference is the key to the transformation of the experiment. In addition, the diversity of basic research proves the urgency of clinical needs. However, there are still few studies that can enter the clinical transformation stage. It is the ultimate goal of each study to translate the results of basic research into clinical practice. As we gradually master the pathogenesis and pathological process, we still hope to solve the problem of diabetes wounds through the treatment of the disease itself, improve the speed and quality of wound healing, to protect the lives of patients, and prevent the occurrence of adverse consequences such as amputation.

## 7 Conclusion

The research between biomaterials and diabetic wounds should be more and more in-depth. However, integrating the above points, there should be a complete idea about the design and utilization aspects of biomaterials. First of all, the selected materials must have excellent biocompatibility. Then, after the pathological conditions of the wound are clarified, these problems should be targeted for direct or indirect regulation, and the microscopic mechanism should be explored under the premise of clear macroscopic effects. The other aspect is to screen suitable materials after analyzing the pathological mechanism of the wound, which seems to be more in line with medical thinking. No matter which idea is chosen, it is certain that the highly adjustable nature of biodegradable biomaterials combined with effective active ingredients can achieve a positive effect in promoting diabetic wound healing. However, for medical researchers, how to apply them more appropriately in clinical practice is another greater challenge.
