# Corrigendum: Effect of composite biodegradable biomaterials on wound healing in diabetes

**DOI:** 10.3389/fbioe.2022.1126860

**Published:** 2023-01-06

**Authors:** Sihang Ren, Shuaichen Guo, Liqun Yang, Chenchao Wang

**Affiliations:** ^1^ NHC Key Laboratory of Reproductive Health and Medical Genetics, Liaoning Research Institute of Family Planning, The Affiliated Reproductive Hospital of China Medical University, Shenyang, China; ^2^ Department of Plastic Surgery, The First Hospital of China Medical University, Shenyang, China; ^3^ The First Clinical College, China Medical University, Shenyang, China; ^4^ Department of Plastic Surgery, The Second Hospital of Dalian Medical University, Dalian, China

**Keywords:** biodegradable biomaterials, diabetic wound, drug delivery systems (DDS), mesenchymal stem cells (MSCs), exosomes

In the published article, there was an error regarding the affiliation for “Sihang Ren.” As well as having affiliations 1, 2 and 3, they should also have affiliation 4, which is as follows: “Department of Plastic Surgery, The Second Hospital of Dalian Medical University, Dalian, China”.

Furthermore, in the published article, there was an error in the **Funding** statement. The Funding statement is not complete. The correct Funding statement appears below.

“This work was sponsored by the Natural Science Foundation of Liaoning Province (2022-YGJC-69) and the support program for excellent young scholars of China Medical University, the National Natural Science Foundation of China (grant/award no. 51872332) and Basic Research Project of the Education Department of Liaoning Province (grant/award no. LJKZ0740).”

The authors apologize for these errors and state that they do not change the scientific conclusions of the article in any way. The original article has been updated.

